# Inter-Island Whole-Genome Comparison Reveals Micro-Evolutionary Dynamics of the Red Fox, Stimulated Through Post-Glacial Sea-Level Alterations

**DOI:** 10.1093/gbe/evaf152

**Published:** 2025-08-21

**Authors:** Takumi Watanabe, Shin Shinojima Satoh, Toshiaki Shiraishi, Shosei Kubota, Yuji Yamazaki

**Affiliations:** Division of Natural Environmental Science, Mount Fuji Research Institute, Yamanashi Prefectural Government, Fujiyoshida, Yamanashi 403-0005, Japan; Graduate School of Science and Engineering, University of Toyama, Toyama, Toyama 930-8555, Japan; Graduate School of Science and Engineering, University of Toyama, Toyama, Toyama 930-8555, Japan; Biotechnological Research Support Division, FASMAC Company Limited, Atsugi, Kanagawa 243-0021, Japan; Curatorial Division, Tateyama Caldera Sabo Museum, Tateyama, Toyama 930-1405, Japan; Biotechnological Research Support Division, FASMAC Company Limited, Atsugi, Kanagawa 243-0021, Japan; Graduate School of Science and Engineering, University of Toyama, Toyama, Toyama 930-8555, Japan

**Keywords:** allopatric evolution, comparative genomics, continental island, demographic dynamics, geographic isolation, Japanese Archipelago

## Abstract

Continental islands provide a system for understanding the mechanisms behind allopatric evolution. The red fox (*Vulpes vulpes*) is characterized by its remarkable dispersibility and adaptability, covering the widest distributional range among the Carnivora. The Hondo red fox (*V. v. japonica*) is a distinctive subspecies that evolved within the Japanese Archipelago, which has an intricate geohistory. Their genomic evolution process among islands offers valuable insights into the relationships between diversification of terrestrial organisms and geographic dynamics associated with climate changes, and conservation of these unique populations. We constructed novel ∼2.4 Gbp whole-genome assemblies with high coverage depth of four wild Hondo red foxes across three predominant islands and estimated the genomic distance, phylogeny, diversity, demography, and split time to reconstruct their biogeographic history at a spatiotemporally fine scale. Despite having a large geographic distance between one another, the pairwise genomic distance was closest between two individuals on the same island. Phylogenetic divergence pattern and runs of homozygosity supported disparate genetic characteristics per island. Historical demographic dynamics exhibited independent trajectories on each island following the Last Glacial, and sudden demographic differentiation was detected during the Hypsithermal. These findings indicate that post-glacial marine transgression degenerated land bridges between islands and strongly contributed to allopatric evolution, even for the highly dispersive generalist. Modern three-island populations are likely considered as respective evolutionarily significant units. This study expands our knowledge regarding the evolutionary history of the red fox and offers crucial insights into the formation process of biodiversity and endemism in terrestrial animals on continental islands.

Significance The Hondo red fox, an endemic subspecies inhabiting three islands in the Japanese Archipelago, faces the risk of local extinction, thereby necessitating the elucidation of their genetic characteristics and population history for conservation. High-throughput sequencing technology is effective for assessing micro-evolutionary dynamics at local, post-glacial, and intra-subspecies levels. This study reveals that the Hondo red foxes on the three islands likely have independent evolutionary histories spanning several millennia and provides insights relevant to conservation for each island population—including genetic diversity, inbreeding, and a historical bottleneck—using whole-genome bioinformatics with a limited sample size. It underscores the significance of incorporating genomic data into biogeographic research to identify potential conservational and taxonomic units by detecting island-specific endemism in the red fox, which was not observed in previous investigations with smaller datasets. Furthermore, the study suggests that a whole-genome strategy is instrumental for non-model and endangered species that are difficult to sample.

## Introduction

**Fig. 1.**
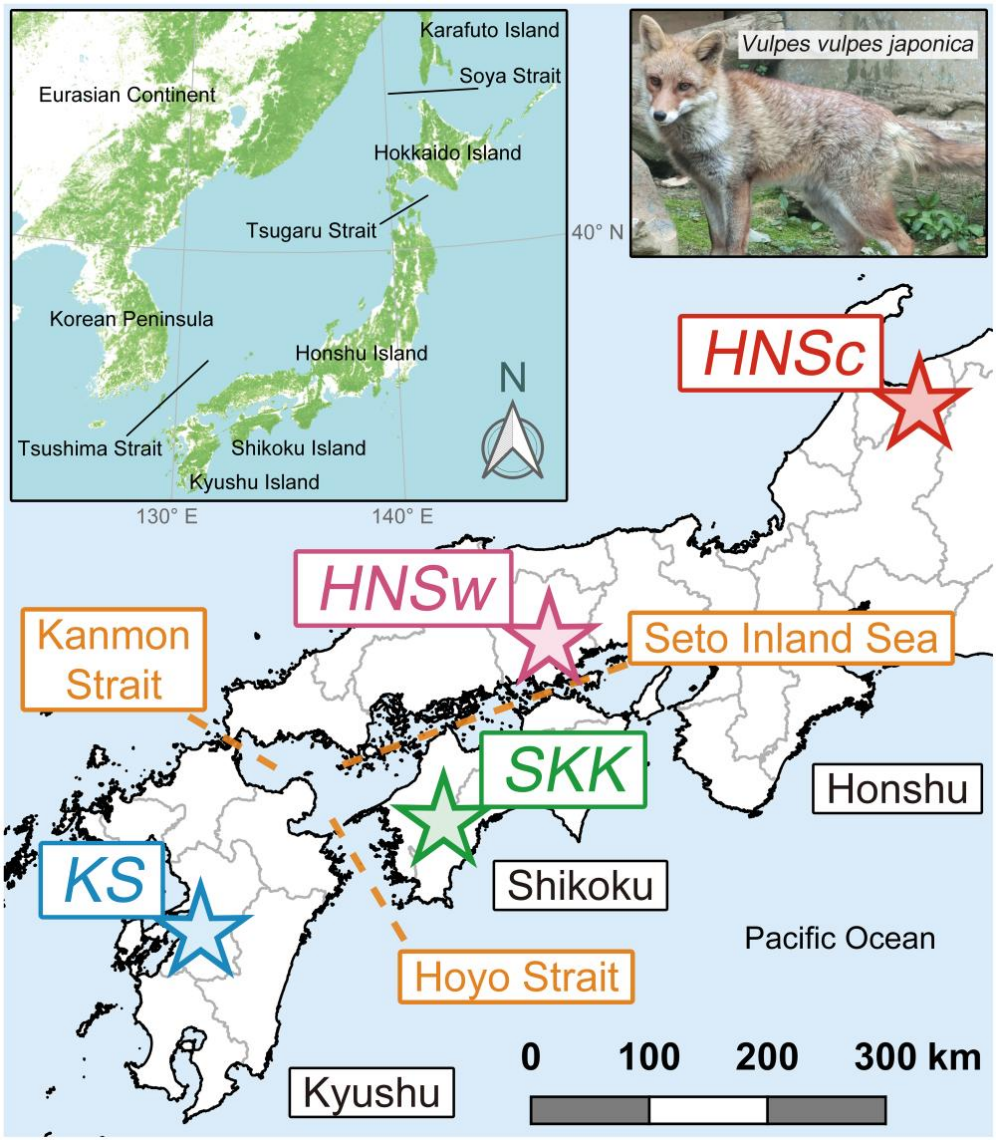
Locality map of the four wild male Hondo red fox samples across three islands. The three broken orange lines indicate straits that are the focus of this study. Gray lines are boundaries of prefectures. The scale bar represents the measure of geographic distance. The inset illustrates biogeographically significant topography around the Japanese Archipelago.

Allopatric evolution is among the most common mechanisms for biological diversification (Lande 1980; Gavrilets 2003; Abbott et al. 2013). Island ecosystems are particularly intriguing for studying drivers underlying the allopatric evolution of terrestrial organisms (Darwin 1859; MacArthur and Wilson 1967; Whittaker and Fernández-Palacios 2007). In biogeography, islands once connected to a continent are categorized as continental islands (Paulay 1994). Continental islands exhibit diverse biota resulting from spatiotemporal variations in colonization and isolation events caused by glacio-eustasy—sea-level fluctuations associated with glacial–interglacial cycles (Heaney 1986; Millien-Parra and Jaeger 1999; Hewitt 2000). The Japanese Archipelago, located in the Far East of the Eurasian Continent, comprises four predominant continental islands: Hokkaido, Honshu, Shikoku, and Kyushu (see inset in Fig. 1). The intricate geohistory and landscape of the Japanese Archipelago have facilitated the allopatric evolution of terrestrial organisms, resulting in the emergence of numerous endemic species, subspecies, and genetic lineages (Dobson 1994; Millien-Parra and Jaeger 1999; Tojo et al. 2017). The Tsugaru and Tsushima straits surrounding the former Paleo-Honshu Island (including the present Honshu, Shikoku, and Kyushu islands) are deep and exhibit significant distributional boundaries for many terrestrial animals (Blakiston 1883; Sato 1969). In contrast, the Seto Inland Sea between the Honshu and Shikoku islands, the Kanmon Strait between the Honshu and Kyushu islands, and the Hoyo Strait between the Shikoku and Kyushu islands are comparatively shallow. The Seto Inland Sea is ∼10 to 40 m deep, involves numerous small islands, and spans a wide range (the Association for the Environmental Conservation of the Seto Inland Sea 2022). The Kanmon Strait is ∼15 m deep and narrow (Yokota et al. 2012), while the Hoyo Strait is ∼100 to 150 m deep (Yoshikawa et al. 1980). The biogeographic effects of these straits on each island's fauna are unclear (Dobson 1994; Millien-Parra and Jaeger 1999). However, as separation of the Honshu, Shikoku, and Kyushu islands likely occurred due to marine transgression during the Holocene Hypsithermal (marine oxygen isotope stage [MIS] 1, ∼9 to 4 ka) following the Last Glacial (MIS 5d to 2, ∼117 to 14 ka) (Ohshima 1990), organisms on each island might have potentially evolved through geographic isolation over the past several thousand years.

Phylogeography is a key research field for reconstructing the evolutionary history of organisms (Avise 2000). Recently, comprehensive genomic data have been incorporated into phylogeographic studies, enabling the robust assessment of fine distributional and demographic dynamics, which was previously challenging to detect by traditional genetic markers like mitochondrial DNA (mtDNA) (e.g. de Ferran et al. 2022; de Jong et al. 2023; Rocha et al. 2023; Quinn et al. 2024). Massive amounts of data from whole-genome sequencing are adequate for estimating population history in silico, even for limited sample sizes (Li and Durbin 2011; Schiffels and Durbin 2014), and offer advantages in studying non-model and rare species. Additionally, as nuclear genomes contain most phenotypic information, identifying their local diversity and endemism is essential for considering taxonomic and conservational units (Funk et al. 2012; Hohenlohe et al. 2021).

The red fox (*Vulpes vulpes* Linnaeus, 1758) is an apex generalist predator characterized by its high adaptability and dispersibility (Trewhella et al. 1988; Dell’Arte et al. 2007; Hisano et al. 2022; Rocha et al. 2023) and possesses the broadest distributional range among the Carnivora (Larivière and Pasitschniak-Arts 1996). The Hondo red fox (*V. v. japonica* Gray, 1868) is an endemic subspecies on the Honshu, Shikoku, and Kyushu islands in the Japanese Archipelago and is taxonomically distinguished from the Kita red fox (*V. v. schrencki* Kishida, 1924) on the Hokkaido Island. Although the Hondo red fox plays a key role in the Japanese ecosystem (Hisano et al. 2022), they have been decreasing recently owing to environmental degradation and listed as a locally threatened or endangered species in certain prefectures (Prefectural Red List: Chiba, 2019; Tokyo, 2020; Kanagawa, 2006; Kyoto, 2021; Osaka, 2014; Fukuoka, 2011; Nagasaki, 2022; Kagoshima, 2015). Watanabe and Yamazaki (2024) aimed to elucidate the phylogeographic process of the red fox in the Japanese Archipelago and estimated the phylogenetic position, spatial genetic structure, time to the most recent common ancestor (tMRCA), and demographic dynamics of the Hondo red fox based on the mtDNA sequence, proposing the following scenario for their population history. An ancestor of the Hondo red fox colonized the Paleo-Honshu Island from the Korean Peninsula via a land bridge generated in the Tsushima Strait during the Penultimate Glacial (MIS 6, ∼191 to 130 ka), and thereafter evolved into a unique monophyletic group in situ through geographic isolation from continental populations. Moreover, their distribution range was divided into the eastern and western parts of the Paleo-Honshu Island from colonization to the Last Glacial Maximum (LGM, MIS 2, ∼23 to 19 ka), and the mtDNA phylogeny diverged into two subclades. The population size and distribution area expanded after the LGM, resulting in secondary contact between the eastern and western ancestral populations. Although the framework for the natural history of the Hondo red fox has been established, the phylogenetic divergences among the Honshu, Shikoku, and Kyushu populations were not observed from the mtDNA marker, and the post-glacial evolutionary history remains unknown. However, the presence of an endemic and frequent haplotype on Shikoku Island (JC03_JD02) and significant differences in the pairwise *F*_ST_ between the Honshu and Shikoku populations suggest a sign of allopatric evolution among the island populations (Watanabe and Yamazaki 2024). To conserve the local genetic endemism of the Hondo red fox, it is necessary to clarify their post-glacial evolutionary history through further surveys harnessing highly sensitive genetic markers from nuclear DNA. This study aimed to assess allopatric evolution among the Honshu, Shikoku, and Kyushu populations of the Hondo red fox based on whole-genome comparisons to reconstruct their phylogeographic history at a spatiotemporally fine scale.

To this end, we comprehensively determined the whole-genome sequences of four wild male Hondo red foxes across the three islands using the next-generation sequencer (NGS) DNBSEQ-G400 (MGI Tech Co., Ltd., Shenzhen, China): central Honshu (*HNSc*), western Honshu (*HNSw*), Shikoku (*SKK*), and Kyushu (*KS*) (Fig. 1 and Table S1). Additionally, we assembled the paired-end short reads into 18 pseudomolecules using a reference-guided approach based on chromosomes of the Tibetan sand fox (*V. ferrilata* Hodgson, 1842) and estimated the genetic diversity, pairwise genomic distance, phylogenetic pattern, demographic dynamics, and population separation history.

## Results

### Genome Assembly Statistics of the Hondo Red Fox

**Fig. 2.**
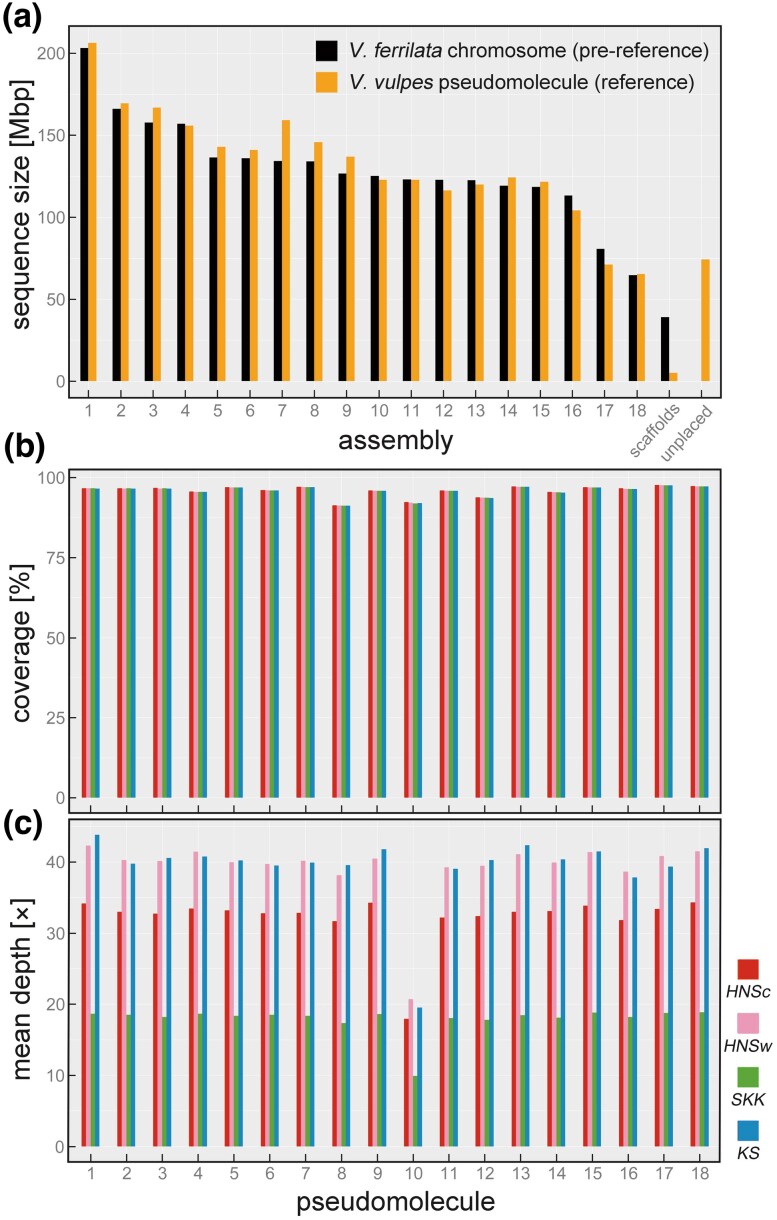
a) Scaffolding of an existing genome resource to create a reference sequence of the red fox. Black and orange represent the pre-reference (chromosome-level assembly of the Tibetan sand fox) and query (82,423 scaffolds of the red fox aligned to the pre-reference) sequences, respectively. Horizontal and vertical axes are fragment number (first to 18th chromosomes or pseudomolecules, other short scaffolds, and unplaced contigs) and sequence length, respectively. The synthesized red fox pseudomolecules were used as the reference genome in this study. b and c) Mapping statistics of the four Hondo red fox genomes. Samples are identified by color. Horizontal axis is pseudomolecule number, whereas vertical axis is (b) coverage against the reference sequence or (c) mean mapping depth.

Since a chromosome-level reference of the red fox was unavailable from databases at the time of analysis, we defined 18 chromosome assemblies from the Tibetan sand fox (GCA_024500485; Lyu et al. 2022) as a pre-reference genome and assigned 82,423 scaffolds from an existing genome resource of the red fox (GCF_003160815; Kukekova et al. 2018) to them to obtain a provisional reference genome. As a result of the scaffolding, the red fox genome assembly comprising 62 synthesized sequences was constructed (Fig. 2a). It contained 18 pseudomolecules homologous to the Tibetan sand fox chromosomes, 42 scaffolds aligned to 216 additional fragments in the pre-reference, one sequence that includes the remaining unplaced contigs, and a mitogenome. The lengths of all the pseudomolecules were approximately the same as those of the chromosomes in pre-reference. The 18 pseudomolecules covered ∼96.8% of the overall data volume. This assembly was used as the reference genome for the present study.

For each sample, NGS generated raw data of ∼71.99 to 123.84 Gbp on ∼527.74 to 827.43 mega reads, ∼87.09% to 92.99% of which were >Q30 (Table S2 and Fig. S1). After quality control (QC), ∼47.92 to 98.83 Gbp on ∼461.21 to 767.83 mega reads were preserved, ∼94.96% to 96.43% of which were >Q30. Data from *SKK* were less abundant than those from the other three samples. Reads of ∼1.25% to 2.90% were excluded from downstream analyses as PCR-duplicate artifacts.

As a result of mapping, the coverage of 18 pseudomolecules was ∼96% in all samples (Fig. 2b and Table 1). The mean depth, excluding the 10th pseudomolecule, was ∼33× for *HNSc*, ∼40× for *HNSw* and *KS*, and ∼18× for *SKK* (Fig. 2c). The mean depth of the 10th pseudomolecule was half that of the other 17. This pseudomolecule was presumed to be a homologous sequence to the X-chromosome, as all samples were male. A synteny analysis of a previous study (Lyu et al. 2022) estimated that the 10th chromosome of the Tibetan sand fox is syntenic to the X-chromosome of the domestic dog (*Canis lupus familiaris* Linnaeus, 1758).

**Table 1:** Whole-genome assembly statistics of the four Hondo red foxes

Sample	Size [bp]	Coverage [%]	Mean depth [×]		Mean *Q*_base_	Mean *Q*_map_	N50		N90		GC [%]
			Autosomal	X-chr.			Count	Length [bp]	Count	Length [bp]	
*HNSc*	2,391,919,281	95.87	33.05	17.9	31.20	57.33	8	140,952,264	16	104,127,256	39.41
*HNSw*	2,391,761,586	95.74	40.28	20.7	33.20	58.06	8	140,940,744	16	104,112,096	39.42
*SKK*	2,394,363,747	95.75	18.35	9.89	30.95	57.15	8	141,144,224	16	104,265,135	39.40
*KS*	2,391,780,000	95.73	40.59	19.5	33.60	58.18	8	140,937,050	16	104,108,982	39.43

Total sequence size, mapping coverage against a reference sequence, mean mapping depths of autosomal and X-chromosomal pseudomolecules, mean base quality score *Q*_base_, mean mapping quality score *Q*_map_, N50, N90, and GC content are presented. The N50 and N90 were calculated from all 62 scaffolds, whereas the other indicators were computed based on 18 pseudomolecules. The mean *Q*_base_ and *Q*_map_ are post-BQSR values.

Across four individuals, 16,246,903 variants were detected against the reference sequence, of which 12,835,477 were single-nucleotide polymorphisms (SNPs) (Fig. S2). All samples shared 5,032,148 SNPs.

A pseudomolecule-level genome assembly of a total of ∼2.4 Gbp was constructed for each sample (Table 1). The mean base quality score *Q*_base_ of the assembly was ∼30.95 to 33.60, and the mean mapping quality score *Q*_map_ was ∼57.15 to 58.18. N50 and N90 based on the overall 62 sequences in the assembly were ∼141 Mbp (eighth segment from the longest) and ∼104 Mbp (16th segment), respectively. The genomic GC content of the Hondo red fox was ∼39.4%.

On the 18 pseudomolecules, 15,634 to 15,678 protein-coding genes, 29 or 30 ribosomal RNA (rRNA), and 243 to 245 transfer RNA (tRNA) were predicted, and the functions of 11,561 to 11,615 genes were annotated (Table S3 and Data TS), of which 10,886 were identified by orthology analysis as single-copy transcripts on the 17 autosomal pseudomolecules. These single-copy transcripts were considered orthologs among samples.

### Genetic Diversity and Inbreeding

The observed heterozygosity rate *H_O_* and Watterson's estimator *θ_W_* (suggesting nucleotide diversity) were at similar levels among all individuals; however, these were relatively lower in *KS* (Table 2). Additionally, the *θ_W_* of *SKK* was slightly higher than that of the others. Runs of homozygosity (RoH) of >1 Mbp based on SNPs after linkage disequilibrium (LD) pruning were observed more frequently and had a larger mean length than RoH based on unpruned data in all samples. This may result from decreased SNP density due to LD-pruning, which reduced the frequency at which segments were broken by heterozygous loci. The two individuals from Honshu Island showed comparable estimates for RoH, while *SKK* had fewer RoH than the Honshu individuals. Notably, *KS* exhibited more frequent RoH than the others, suggesting inbreeding in recent periods. Overall, although the levels of genetic diversity did not vary significantly among the samples, RoH suggested different inbreeding levels per island.

**Table 2:** Genetic diversity of the four Hondo red fox genomes

Sample	*H_O_*	*θ_W_* (×10^−3^)	RoH (overall)			RoH (post-LD-pruning)		
			Count	Total size [Mbp]	Mean size [Mbp]	Count	Total size [Mbp]	Mean size [Mbp]
*HNSc*	0.33	4.54	1	1.05	1.05	46	92.22	2.00
*HNSw*	0.31	4.47	1	1.03	1.03	46	75.43	1.64
*SKK*	0.33	4.73	0	0.00	0.00	9	13.74	1.53
*KS*	0.28	4.43	10	14.18	1.42	135	268.32	1.99

Observed heterozygosity rate *H_O_*, Watterson's estimator *θ_W_*, and runs of homozygosity (RoH) based on overall and post-LD-pruning SNPs are presented. The indicators were computed from 17 autosomal pseudomolecules using PLINK v.1.90b6.21 and ANGSD v.0.940.

### Phylogenomics and Evolutionary Distance Between Individuals

**Fig. 3.**
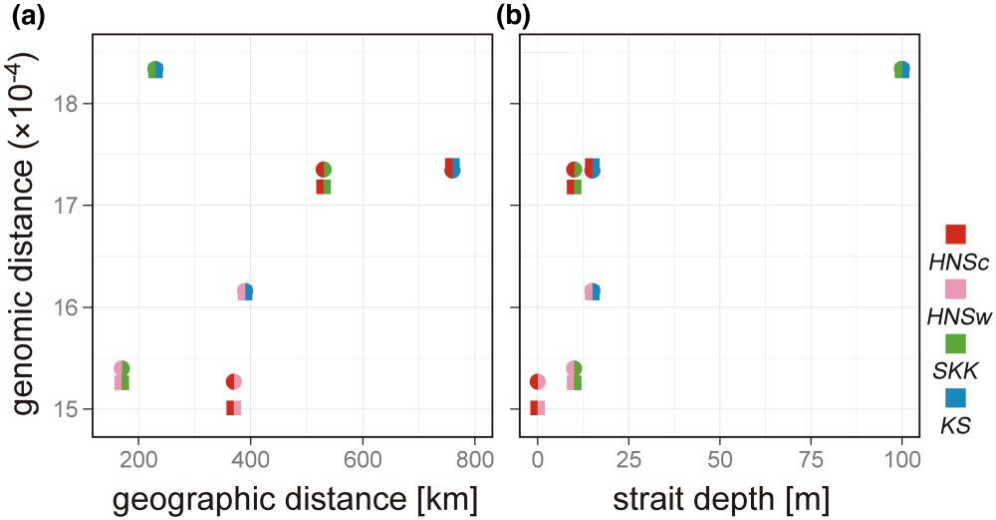
Relationship between pairwise genomic distance and (a) Euclidean geographic distance or (b) strait depth in the four Hondo red foxes across three islands. Sample pair is identified by color. The genomic distance was computed from 18 pseudomolecule assemblies of ∼2.4 Gbp based on Hamming (circle) and MinHash (square) methods using Skmer v.3.3.0 and Mash v.2.3, respectively. Depths of the Seto Inland Sea, Kanmon Strait, and Hoyo Strait are roughly assumed to be ∼10, ∼15, and ∼100 m, respectively, based on their shallowest points.

Pairwise genomic distances among samples were closest between the two Honshu individuals, second closest between *HNSw* and *SKK*, third closest between *HNSw* and *KS*, and farthest between *SKK* and *KS* in both Hamming and MinHash calculation methods (Table S4). The two distances between *HNSc* and *SKK*, and *HNSc* and *KS* were almost the same level, and the second farthest among the sample pairs. All MinHash values were significant (*P* < 0.01). Proportionality between genomic and Euclidean geographic distances was not observed (Fig. 3a), whereas that between genomic distance and strait depth was suggested (Fig. 3b). However, there is a degree of uncertainty in these results due to the limited sample size.

**Fig. 4.**
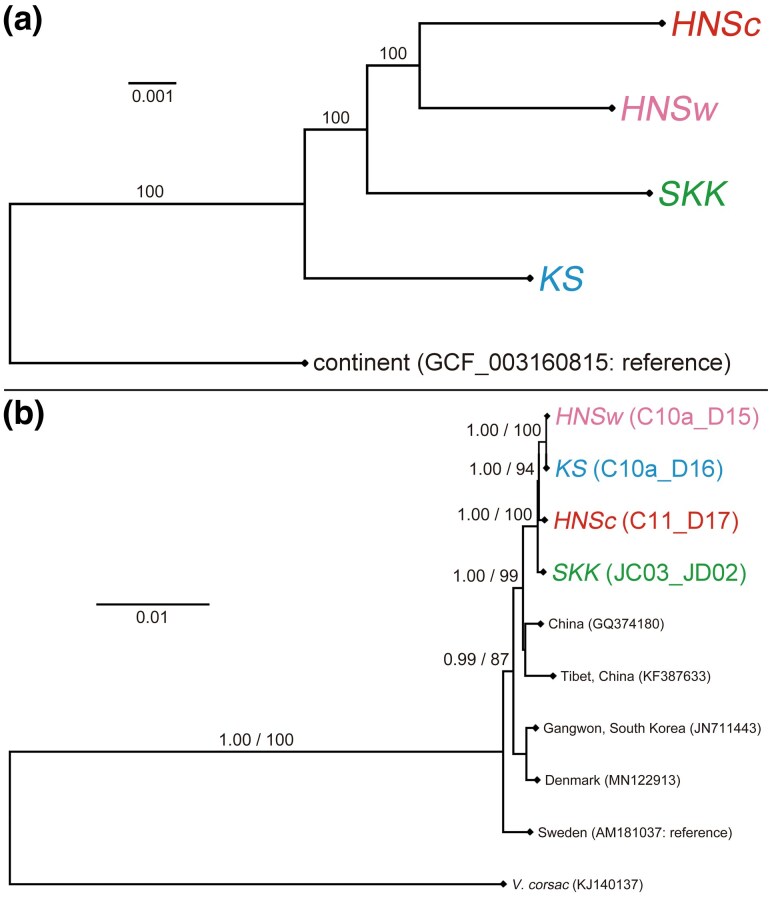
a) Genomic phylogenetic tree of the Hondo red fox based on a concatenated sequence of 10,886 orthologous transcripts (16,221,029 bp) on 17 autosomal pseudomolecules. The tree was estimated by the maximum likelihood method using RAxML-NG v.1.2.0. Bootstrap value based on 100,000 iterations is denoted above the branch. The outgroup is a continental red fox from a Russian experimental farm (Kukekova et al. 2018). The scale bar represents the measure of genetic distance. b) Mitogenome phylogeny of the red fox based on a 15,467-bp sequence across 37 transcripts. The tree was estimated by the Bayesian and maximum likelihood methods using MrBayes v.3.2.7 and RAxML v.8.2.10. Support rate (posterior probability/bootstrap value) is denoted above the branch. Haplotype name, which was defined by Watanabe and Yamazaki (2024), is shown following the sample name.

For a phylogenetic analysis, the orthologs were concatenated to generate a 16,221,029-bp alignment. The genomic phylogenetic tree based on a maximum likelihood method exhibited the following topology: divergence between *KS* and the common ancestor of the other three individuals, followed by divergence between *SKK* and the ancestor of the Honshu individuals (Fig. 4a). Despite geographic remoteness between one another, *HNSc* and *HNSw* were monophyletic. The branches for all the clades were supported by 100% bootstrap values. Thus, the results suggested that the Hondo red fox forms independent genetic groups on each island. In a phylogenetic estimation based on concatenated SNP loci comprising 7,228,522 variant sites, significant differences were detected in base composition among sequences by the *χ*^2^ test (*P* < 0.05); consequently, performing a reliable analysis was not possible (Fig. S3). This is likely because the sample size was too small for a relative analysis based solely on variant sites.

The topology between the maximum likelihood and Bayesian trees based on a 15,467-bp mitogenome coincided with one another, and four haplotypes from the Hondo red fox were monophyletic with 100% support rates (Fig. 4b). Divergence patterns among samples differed between the genomic and mitogenomic trees, with the samples not clustering by island in the mitogenomic tree. Specifically, *HNSw* was placed as a sister to *KS*, and *HNSc* was a sister to the *HNSw–KS* pair in the mitogenomic tree. This discrepancy might have resulted from the differences in the amount of data, modes of inheritance, and mutation rates (Rubinoff and Holland 2005). Additionally, sex-biased dispersal, secondary contact, ancestral polymorphisms, and genetic recombination of nuclear DNA might have resulted in the different tree topologies between nuclear and mitochondrial DNA (Toews and Brelsford 2012; Watanabe and Yamazaki 2025).

In both principal component analysis (PCA) plots based on 12,248,003 SNPs from 17 autosomal pseudomolecules and 489,033 post-LD-pruning SNPs, the contribution rates were comparable in the PC1 to PC3 axes (all dimensions); therefore, it was not possible to observe genetic similarity among samples (Fig. S4). Similarly, in the ancestral clustering analysis, cross-validation error was lowest in the number of ancestral clusters *K* = 4 (per sample), and evaluating admixture among individuals was not feasible (Fig. S5). Overall, the sample size was insufficient to conduct SNP-based inter-individual analyses, likely because they are relative computations among samples.

### Demography and Population Differentiation Between Islands

**Fig. 5.**
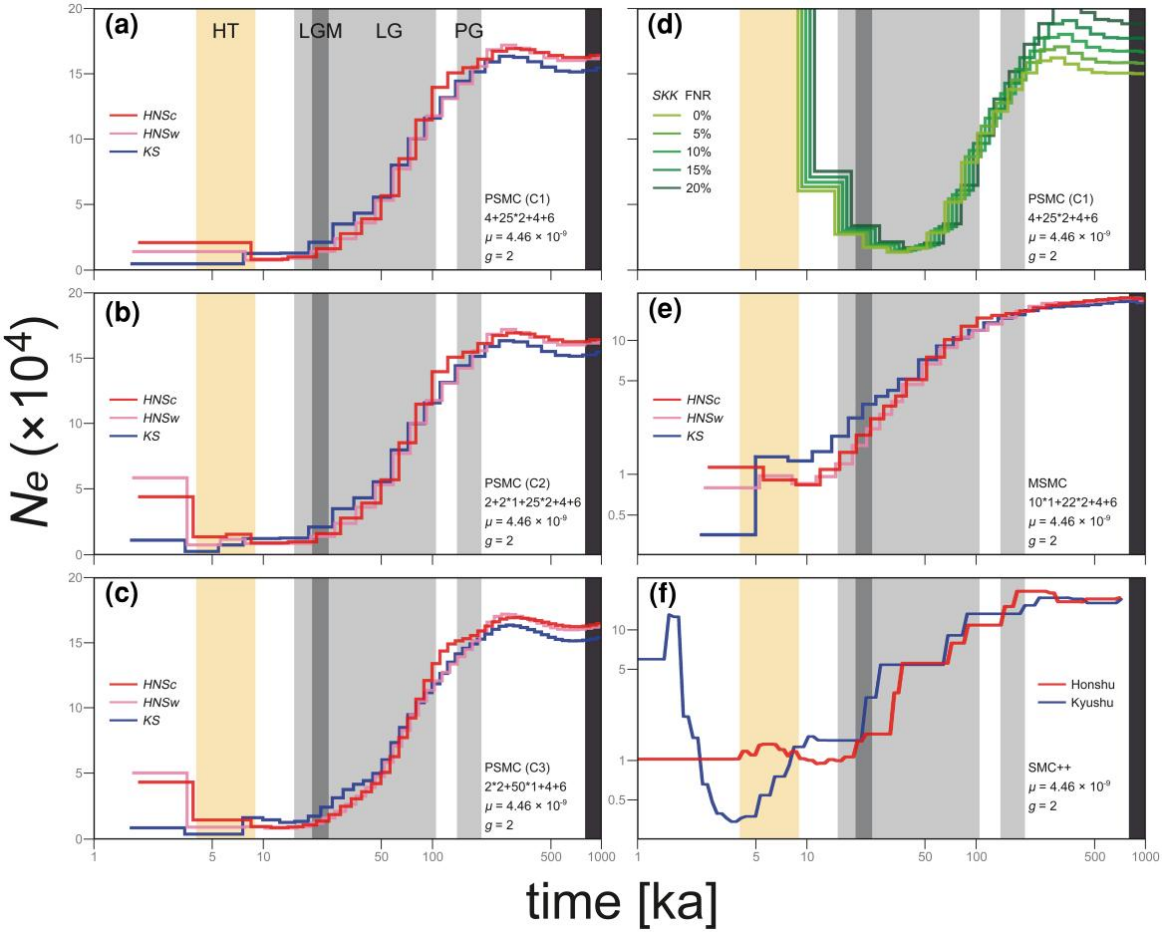
Historical demographic dynamics in ancestral populations of the four Hondo red fox individuals. Horizontal and vertical axes show a logarithmic timeline and effective population size *N_e_*, respectively. Samples or populations are coded by line colors. Gray and orange backgrounds represent glacial and post-glacial warm periods, respectively (PG: Penultimate Glacial, LG: Last Glacial, LGM: Last Glacial Maximum, and HT: Hypsithermal). The black right edge of each panel indicates the approximate speciation time (∼1000 to 800 ka) between the red fox and Rüppell's fox (*V. rueppellii* Schinz, 1825) (Basuony et al. 2023). Estimation model, time-segment pattern, genomic mutation rate *µ* (per site/generation), and generation time *g* are detailed at the bottom right corner in each panel. Results of bootstrap iterations for all simulations are provided in Figs. S6 to S9. a to c) PSMC plots of the Honshu and Kyushu populations based on three temporal segment patterns (conditions C1 to C3); estimated using diploid consensus assemblies of 17 autosomal pseudomolecules. d) PSMC plots of the Shikoku population with false negative rate (FNR) correction of heterozygosity applied in varying degrees. e) MSMC plots of the Honshu and Kyushu populations; estimated using mapping reads on 17 autosomal pseudomolecules. f) SMC++ plots of the Honshu and Kyushu populations; estimated using variant data on 17 autosomal pseudomolecules. Samples *HNSc* and *HNSw* were integrated into the Honshu population. Vertical axis is logarithmic in panels (e) and (f).

In the pairwise sequentially Markovian coalescent (PSMC) simulations (Li and Durbin 2011) based on three time-segment patterns (conditions C1 to C3), the resolution of demographic reconstruction in the post-glacial stage was higher in C2 and C3 (Fig. 5a to c). Results from 100 bootstrap permutations converged with a result from the whole-genome in all samples (Figs. S6 to S8). Despite independent analyses for each individual, the demographic trajectories of all samples were consistent until the Last Glacial, indicating common evolutionary backgrounds and reproducibility of simulations. The effective population size *N_e_* of Hondo red fox ancestors was estimated to decrease continuously from the Penultimate Glacial to the Last Glacial, and no other distinctive demographic signals were detected. Following the Last Glacial, population growth around the Hypsithermal was observed from the two Honshu individuals, whereas *N_e_* from *KS* declined or stagnated, suggesting disparate population histories between the Honshu and Kyushu islands in the post-glacial stage. False negative rate (FNR) correction of heterozygosity was applied to only *SKK* given its <30× sequencing depth (Fig. 5d). However, ancestral *N_e_* from *SKK* expanded unnaturally after the Last Glacial, and it was not modified by FNR correction. This may represent a limitation of PSMC using a sample with insufficient coverage depth (Nadachowska-Brzyska et al. 2016).

**Fig. 6.**
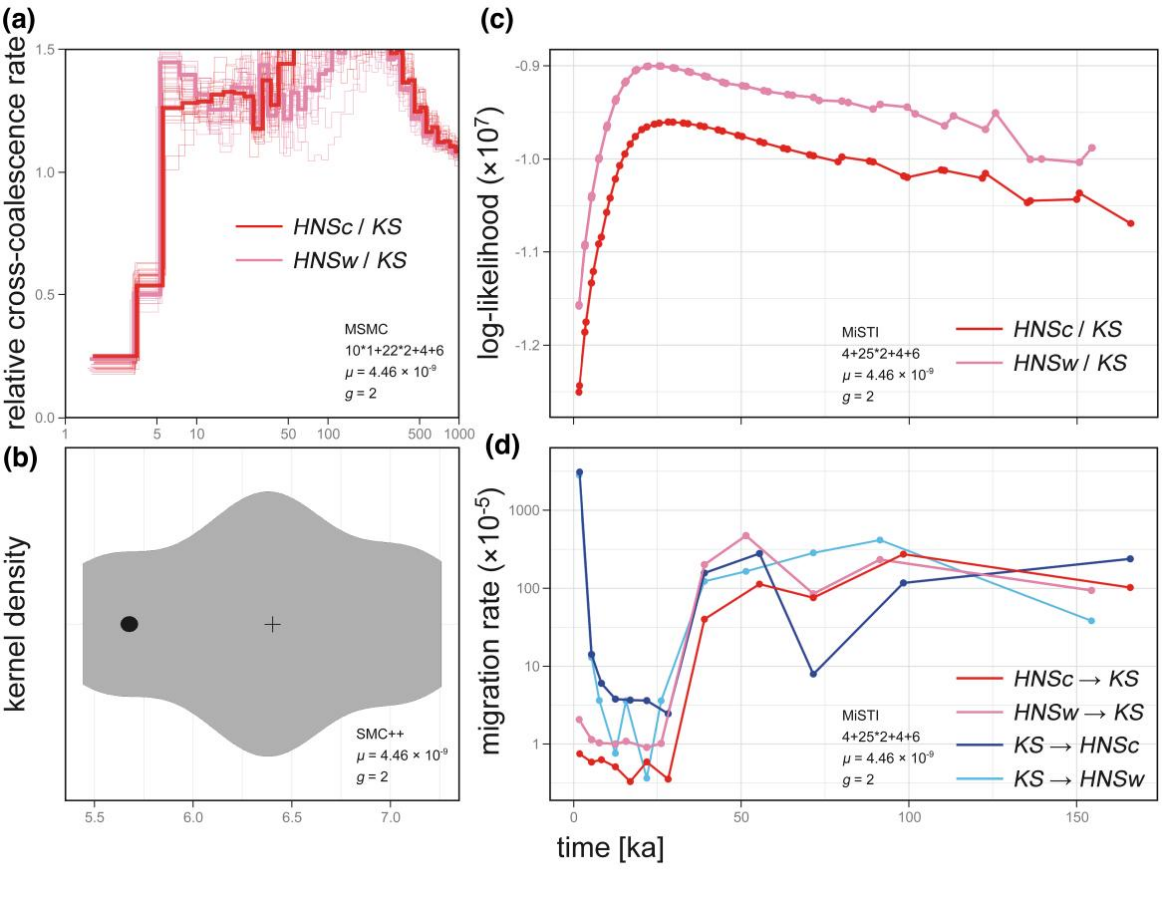
Demographic differentiation chronology between the Honshu and Kyushu populations in the Hondo red fox, based on the coalescent theory and site frequency spectrum. The Honshu population includes two diploid genomes (*HNSc* and *HNSw*), whereas the Kyushu population includes one genome (*KS*). Horizontal axis is a timeline. Estimation model, time-segment pattern, genomic mutation rate *µ* (per site/generation), and generation time *g* are explained in each panel. a) Population separation history estimation using MSMC. Vertical axis indicates the relative cross-coalescence rate; a value approximating zero suggests demographic divergence. A dark line is based on whole genomes, whereas 20 light lines are based on bootstrap permutations. b) Split time estimation using SMC++. The violin plot represents the probability density distribution for genome split times based on 20 bootstrap permutations, and a cross denotes their median. The black circle is a point estimator based on whole genomes. c) Split model test using MiSTI. Log-likelihood was computed for 50 split-time scenarios between the two genomes using the results of PSMC C1. d) Migration rate inference using MiSTI. For split models with the highest log-likelihood, optimized bidirectional migration rates in 12 time intervals are plotted. Vertical axis is logarithmic in this panel.

The demographic simulation using the multiple sequentially Markovian coalescent (MSMC) algorithm (Schiffels and Durbin 2014) supported nearly identical dynamics to the results of PSMC (Fig. 5e). Specifically, the ancestral *N_e_* based on two Honshu individuals increased or stabilized after the Last Glacial, whereas that based on a Kyushu individual decreased. Results from 20 bootstrap reconstructions confirmed this difference (Fig. S9a to c). The relative cross-coalescence rate, which reflects population separation history, between Honshu and Kyushu populations abruptly dropped toward zero at ∼5 ka in both comparisons of *HNSc*/*KS* and *HNSw*/*KS*, suggesting the cessation of coalescence between the populations and the demographic differentiation up to the present time (Fig. 6a). Bootstrapping reproduced these results.

SMC++ (Terhorst et al. 2017) based on site frequency spectrum (SFS) and LD information (it is more effective than PSMC-based methods for estimation in recent periods) exhibited similar demographic dynamics as PSMC and MSMC (Fig. 5f). Notably, this model clearly detected a bottleneck of the Kyushu population in the post-glacial stage. The results of 20 bootstrap iterations supported this bottleneck event (Fig. S9d and e). Additionally, the split time between Honshu and Kyushu populations was estimated to be 5,678 years ago (*N_0_* = 11,210.76, coalescent split value = 0.1266) from whole-genome data (Fig. 6b). Split times from bootstrap data were in the range of 7,258 to 5,444 years ago, and their median was 6,403 years ago. These results correspond with the dynamics of the relative cross-coalescence rate in MSMC.

In MiSTI inference (Shchur et al. 2022), using the results of PSMC, split scenarios between Honshu and Kyushu populations about 28,239 years ago (*HNSc*/*KS*: time interval 20) and 25,598 years ago (*HNSw*/*KS*: time interval 19) had the highest log-likelihood (Fig. 6c). These split times correspond to the LGM and are older than the divergence time estimated by MSMC and SMC++. In these split scenarios, bidirectional migration rates declined suddenly around the LGM, suggesting a limitation of gene flow between the populations (Fig. 6d). Gene flow dynamics between the populations was roughly symmetric. Overall, the coalescent analyses suggested disparate population histories and demographic differentiation on each island during the post-glacial stage.

## Discussion

This study provides the first whole-genome assembly of the wild Hondo red fox at a pseudomolecule level with high coverage depth and contributes to our understanding of the genomic evolution of terrestrial organisms across geographically proximate continental islands. The comparative analyses utilizing massive genomic data elucidated the allopatric micro-evolution of the Hondo red fox on each island associated with post-glacial climate changes, which was not revealed by the mtDNA phylogeography, and detected a front line of the formation process of biodiversity and endemism. Additionally, valuable insights into the conservation of this unique subspecies were obtained. Meanwhile, some shortcomings of whole-genome analysis using small sample sizes became apparent. This study underscores the advantages and limitations of whole-genome bioinformatics in biogeography that focuses on spatiotemporally fine-scale evolutionary history.

### Common Evolutionary Background Until the Last Glacial

Despite estimating the demographic histories for each individual independently using PSMC, all demographic trajectories were consistent up to the Last Glacial. Moreover, the relative cross-coalescence rate in MSMC was over 1.0, and the migration rates in MiSTI were high until the Last Glacial. These results support that the Hondo red foxes on the three islands share a common evolutionary history as a monophyletic group descended from a single ancestral population, coinciding with the previous mtDNA phylogeography (Watanabe and Yamazaki 2024) and the mitogenome tree in the present study. Additionally, the absence of other demographic signals until the Last Glacial, except for a continuous decline, suggests no evolutionary events that might increase the heterozygosity of the ancestral population, such as additional colonization after an initial opportunity or interaction with other populations. This result aligns with a single-time colonization and high endemism of the Hondo red fox proposed by the previous mtDNA phylogeography (Watanabe and Yamazaki 2024). Overall, the findings from whole-genome marker supported the phylogeographic hypothesis from the previous mtDNA study without contradiction.

In the previous mtDNA study, the Bayesian divergence time estimation indicated that the tMRCA of the Hondo red fox was ∼148 (95% HPD: 236 to 80) ka, and their colonization occurred during the Penultimate Glacial (Watanabe and Yamazaki 2024). Geological evidence supports that the last land bridge in the Tsushima Strait has degenerated following the Penultimate Glacial (Ohshima 1990; Park et al. 2000). Therefore, the *N_e_* attenuation since the Penultimate Glacial in PSMC, MSMC, and SMC++ likely represents the founder effect by colonization and isolation on the Paleo-Honshu Island (Frankham 1996; Waters et al. 2013). In Eurasian red foxes, because similar mtDNA haplotypes (Holarctic clade) are distributed widely from Europe to East Asia (Kutschera et al. 2013; Statham et al. 2014), an enormous population size with broad gene flow is expected. In contrast, the Hondo red fox has been isolated within the Paleo-Honshu Island. Thus, the historical *N_e_* of the Hondo red fox was likely much smaller than that of the continental population, and the colonization event might have been detected as a demographic decrease. In a recent study involving MSMC simulation for a red fox sample from the Yamal Peninsula in Russia (Quinn et al. 2024), the ancestral *N_e_* of the Siberian population corresponded with that of the Hondo red fox before the Penultimate Glacial (∼15 × 10^4^), but the subsequent rapid demographic falloff was not observed; the subsequent *N_e_* of the Siberian population remained obviously higher than that of the Paleo-Honshu population. Therefore, the *N_e_* decline did not likely occur in the Eurasian populations and was specific to the Hondo red fox ancestors. This supports that the *N_e_* falloff resulted from colonization and isolation in Japan. Additionally, the estimated *N_e_* of several American populations consistently decreased and differentiated from that of the Eurasian population after ∼400 to 300 ka (Quinn et al. 2024), when the red fox first colonized the North American Continent via a land bridge formed in the Bering Strait (Sacks et al. 2018). This case resembles the historical *N_e_* dynamics of the Hondo red fox, suggesting common evolutionary signals regarding the colonization process beyond the sea in this species.

### Divergence of Population History Through Post-glacial Climate Change

A proportional relationship between pairwise genomic distances and geographic distances among samples was not observed, whereas that between genomic distances and strait depths was suggested. Specifically, the genomic distance was closest between two land-connected individuals on the Honshu Island despite their geographic remoteness and was farthest between *SKK* and *KS* despite their geographic closeness, as they are separated by the deep Hoyo Strait. Additionally, the genomic phylogenetic tree supported that samples were genetically clustered on each island with high bootstrap values. The RoH also exhibited different tendencies for each island. Therefore, straits have likely acted as strong dispersal barriers, even for the highly dispersive generalist, and contributed to the genetic differentiation of each island population. The highest genomic distance between *SKK* and *KS* may reflect that gene flow was restricted between the Shikoku and Kyushu populations by forming the Hoyo Strait earlier than gene flow between other populations, suggesting fine evolutionary dynamics associated with post-glacial sea-level alterations. A larger sample size may improve our understanding of the relationships between bathymetric topography and genomic diversification patterns on continental islands through statistical analyses, such as the Mantel test.

Based on the PSMC, MSMC, and SMC++ results, we observed the population growth on the Honshu Island after the Last Glacial. This demographic pattern aligns with a Bayesian skyline plot based on mtDNA in the previous study (Watanabe and Yamazaki 2024). However, the ancestral *N_e_* of *KS* plummeted in the post-glacial stage and differed from that of the Honshu individuals. Although this difference seems small, the convergence of bootstrap permutations provides reliability. Therefore, the Honshu and Kyushu populations likely have disparate evolutionary histories after the Last Glacial. Given that the Honshu Island (∼227,940 km^2^) is considerably larger than the Kyushu Island (∼36,780 km^2^), the abundance and distribution range of the Honshu population have likely expanded, whereas those of the Kyushu population might not have. Additionally, a secondary contact between eastern and western ancestral populations on the Paleo-Honshu Island (Watanabe and Yamazaki 2024) might have increased the genetic diversity of the Honshu population, leading to the higher *N_e_* estimates (Abbott et al. 2013).

The relative cross-coalescence rate in MSMC and split-time estimation in SMC++ indicated demographic differentiation between the Honshu and Kyushu individuals during the post-glacial period, especially the Hypsithermal. This age is characterized by rapid marine transgression owing to deglaciation (Cronin 2012). A geological study proposed the formation of the Kanmon Strait at ∼5 ka (Ohshima 1990), which aligns with the results of MSMC and SMC++. Therefore, the post-glacial sea-level change separated the Honshu and Kyushu islands by degenerating land bridges, likely contributing to the genetic diversification of the Hondo red fox. However, MiSTI inferred that the migration rates between the Honshu and Kyushu populations declined around the LGM, and it differed from the MSMC and SMC++ estimates. This may be due to the unphased genomes (maternal and paternal haploid sequences were not distinguished) or an insufficient resolution of the PSMC-related algorithm in recent periods. Additionally, as a large river may have existed in the current Kanmon Strait during the LGM (Yoshikawa 1953), the population splitting in the context of migration might have extended to before the post-glacial island segmentations. Based on the results of MSMC, the demographic trajectory of *KS* slightly deviated from those of *HNSc* and *HNSw* following the LGM, suggesting that the population differentiation gradually began prior to the island separations. Further examinations are necessary to address the discrepancies in split times, yet all analyses support that the Honshu and Kyushu populations have different demographic histories after the island separation, at least. Although investigations on genomic phylogeography of other Japanese mammals are limited, a previous study on the Japanese black bear (*Ursus thibetanus japonicus*) suggested that demographic histories based on two whole genomes from Honshu and Shikoku islands diverged within ∼30 ka (Kishida et al. 2022). This inference aligns with the results of the present study, suggesting potential variations among modern faunas at a genomic level on the three islands. This finding serves as a valuable case for the relationship between the genomic evolution of terrestrial organisms and the intricate geohistory of continental islands associated with climate changes.

A recent study using genome-wide SNPs from reduced-representation sequencing revealed that the genetic characteristics of the red fox on the British Isles differentiated from those on the European Continent during the post-glacial period (McDevitt et al. 2022). Additionally, genetic differentiation was also observed between Great Britain and Ireland (McDevitt et al. 2022). These fine genetic structures were not detected by mtDNA phylogeography due to the limited data size (Teacher et al. 2011). The findings of the present study align with this case, suggesting the parallel phylogeographic processes of the red fox on many continental islands through the post-glacial sea-level rise, which are difficult to elucidate using traditional genetic markers. Further genomic studies in the Hokkaido–Karafuto (Sakhalin)–Chishima (Kuril) island system, which is biogeographically distinguished from the Honshu–Shikoku–Kyushu system (Dobson 1994; Millien-Parra and Jaeger 1999), likely offer valuable knowledge into the evolutionary dynamics of red fox on continental islands, including the assessment of potential ancient gene flow between the Hondo and Kita red foxes.

In the previous mtDNA phylogeography of the Hondo red fox, the eastern and western subclades were distributed sympatrically in central Honshu, and the genetic distance between these subclades was much greater than that among the islands (Watanabe and Yamazaki 2024). Despite the anticipation that the genome of *HNSc* may be genetically affected by the eastern ancestral population on the Paleo-Honshu Island, this study did not observe such a distinct sign. Specifically, the pairwise genomic distances between *HNSc* and other samples were smaller than the distance between *SKK* and *KS*, and *HNSc* was placed in the most internal clade in the genomic phylogenetic tree. This suggests that the spatial genetic structure resulting from historical east–west vicariance was not as clearly maintained in the nuclear DNA as in the mtDNA. Male-biased dispersal patterns of the red fox (Walton et al. 2021) might have attenuated the genetic population structure across the Paleo-Honshu population (Hirata et al. 2017; Tanaka et al. 2020). A recent patrilineal phylogeographic study of the Hondo red fox using polymorphic microsatellite markers on the Y-chromosome revealed a phylogenetic divergence into two major clades (like mtDNA) and their ill-defined spatial structures (unlike mtDNA), supporting the male-mediated gene flow hypothesis (Watanabe and Yamazaki 2025).

### Insights into the Conservation of the Hondo Red Fox

Overall, the findings of this study suggest that each island population of the Hondo red fox should be considered as independent evolutionarily significant units with respective genetic endemism and future evolutionary potentials. These populations should not be admixed by anthropogenic introduction, and in-situ conservation must be addressed to prevent local extinctions and genetic disturbances, especially in Shikoku and Kyushu populations. Although direct comparisons may be difficult due to variations in markers and calculation methods among studies, the *H_O_* of the Hondo red fox may be lower than that of Eurasian populations (∼0.5 to 0.6) and similar levels to that of certain North American populations (Sacks et al. 2011; Statham et al. 2014, 2018). The conservation genetics of several American populations, such as mountainous isolated subspecies, is well-documented (Sacks et al. 2011; Preckler-Quisquater et al. 2024; Quinn et al. 2024). However, as genetic assessments of the Hondo red fox remain insufficient, further information is required to develop their appropriate conservation plans.

The sequencing depth of *SKK* was low compared to the other individuals. Low depth typically results in false negatives in heterozygosity calls due to the lack of alleles, leading to high homozygosity (Nielsen et al. 2011). Additionally, because it is thought that the population size of the Hondo red fox was small on the Shikoku Island until the 20th century (Inaba 2018), low genetic diversity was expected. Nevertheless, *SKK* had higher *H_O_* and *θ_W_* than the Honshu individuals and fewer RoH. These results suggest that artificial introduction, performed to improve the abundance in numerous municipalities of Shikoku during the 20th century (Inaba 2018), affected their genetic diversity. Although the source of the introduced exotic red foxes was unidentified (Inaba 2018), this study and the previous mtDNA study (Watanabe and Yamazaki 2024) suggest that they were the Hondo red fox on Honshu or Kyushu islands, not Kita or continental red foxes. As this anthropogenic introduction may have damaged the local genomic endemism of the Shikoku population, even within the same subspecies, further genetic assessments with more samples are warranted.

The Hondo red fox is listed on the Prefectural Red List of certain prefectures in Kyushu (Fukuoka, 2011; Nagasaki, 2022; Kagoshima, 2015). The Kyushu population likely possesses high endemism owing to its independent population history across several millennia. However, the genome of *KS* exhibited low genetic diversity compared to the other individuals and had numerous RoH regions, suggesting a sign of inbreeding. Additionally, SMC++ revealed a demographic bottleneck in the Kyushu population during the post-glacial stage. This bottleneck event may have resulted from a catastrophic eruption of the Kikai Caldera supervolcano, located in the south of Kyushu Island, at ∼7,300 years ago. It was the world's largest eruption in the Holocene, covering the southern part of the Kyushu Island with pyroclastic flow (Shimizu et al. 2024). The bottleneck effect might have enhanced genetic differentiation of the Kyushu population through acute genetic drift (Wright 1931). Given that the Kyushu population may be genetically endemic yet vulnerable, continuous monitoring of their abundance and genetic diversity is necessary for conservation. To avoid genetic disturbances, artificial introductions from the Honshu or Shikoku populations should not be undertaken unless local extinction becomes an imminent threat.

Although this study provided valuable findings by assembly-based analyses, several concerns remain due to the small sample size. First, each sample may not be representative of its corresponding island population, leaving uncertainty in interpreting the results. Second, as the sample size was insufficient for performing SNP-based inter-individual analyses, we could not investigate the gene flow dynamics, such as admixture. Third, as the PSMC-based approaches are not very sensitive in detecting recent demography, the post-glacial results should be further examined using another method, such as an SFS- or LD-based demographic reconstruction or a population history test by approximate Bayesian computation. Fourth, as the present analyses focused on only neutral evolution, the genetic bases of morphological and physiological adaptations were not assessed. Future studies utilizing numerous samples and genome-wide SNPs from reduced-representation sequencing or low-coverage whole-genome resequencing may resolve these issues. To facilitate such research, this study provides fundamental knowledge and resources, including the first wild-type genome assemblies of the Hondo red fox.

## Conclusions

This study provides valuable genome resources from wild red foxes on continental islands and offers significant insights into the formation processes of diversity and endemism in terrestrial organisms. The various results from the whole-genome analysis enhanced and extended the phylogeographic hypothesis of the Hondo red fox, which has been proposed by the previous mtDNA study. The relationship between genomic distance and strait depth, the phylogenetic pattern, variation of RoH, independent demography after the Last Glacial, and sudden differentiation during the Hypsithermal support the allopatric micro-evolution among the Honshu, Shikoku, and Kyushu populations, stimulated by the complex geohistory of continental islands associated with post-glacial climate change. Straits acted as crucial dispersal barriers even for this highly dispersive and adaptable generalist, and the dynamic eustatic movement drove their genetic diversification at the local, post-glacial, and intra-subspecific levels. Modern Hondo red fox populations on the three islands likely have disparate genetic characteristics and evolutionary potentials, and this finding should be considered in the conservation of their local genetic endemism. The red fox may serve as a model for understanding recent evolutionary dynamics of terrestrial carnivores on continental islands.

## Materials and Methods

### Sample Selection

Muscle tissue samples of four wild male Hondo red foxes across the three islands, collected ethically by the previous study (Watanabe and Yamazaki 2024) during 2013 to 2018, were selected for whole-genome shotgun sequencing (WGS): central Honshu (*HNSc*), western Honshu (*HNSw*), Shikoku (*SKK*), and Kyushu (*KS*) (Fig. 1 and Table S1). All samples belonged to the western (HNb) subclade in the mtDNA phylogeny. The map of Fig. 1 was generated using QGIS v.3.28.2 (QGIS Association, Switzerland), the administrative district data in the digital national land information repository, and the vegetation (percent tree cover) data in the Geographical Survey Institute Tiles, provided by the Ministry of Land, Infrastructure, Transport, and Tourism in Japan.

### Whole-Genome Sequencing

Genomic DNA (gDNA) was extracted from each sample using an automatic nucleic acid purification device Maxwell RSC Instrument (Promega Corp., Madison, WI, U.S.A.), following the protocol of the Maxwell RSC Tissue DNA Kit. Tissues were cut, placed in 120 µL Tris–ethylenediaminetetraacetic-acid buffer, digested with 12 µL proteinase K solution for 3 h at 56 °C, and homogenized using pestles. Each 100 µL sample suspension was injected into a cartridge of the extraction kit. Purified gDNA solutions were sent to a laboratory at FASMAC Co., Ltd. (Atsugi, Japan) for WGS.

Library preparation was conducted based on the protocol of the KAPA Hyper Prep Kit (Kapa Biosystems Inc., Wilmington, MA, U.S.A.), after gDNA quantification using an Invitrogen Qubit 4 Fluorometer (Thermo Fisher Scientific Inc., Waltham, MA, U.S.A.) and subsequent dilution to 10 ng/µL. The gDNA was physically sheared through sonication using Picoruptor 2 (Diagenode S.A., Seraing, Belgium), followed by end-repair and dA-tailing. After adapter ligation and magnetic bead purification, four cycles of PCR amplification were performed. The insert size distribution was measured through gel electrophoresis using an Agilent 2100 Bioanalyzer (Agilent Technologies Inc., Santa Clara, CA, U.S.A.). Size selection was performed based on magnetic bead adsorption after pooling the samples.

We adopted the DNBSEQ as a massively parallel sequencing method. Five cycles of adapter-conversion PCR were performed to incorporate bridge regions. Following library circularization, a DNA-ball (DNB) was generated through rolling-circle replication. DNBs were injected into the flow cell of the DNBSEQ-G400, and paired-end short reads of 150 bp each were comprehensively sequenced. Adapter contamination was eliminated during data output. Raw read sequences were deposited in the International Nucleotide Sequence Database (INSD) (accession number: DRR530053–DRR530056).

### Data Quality Control

The quality of the raw data was assessed using FastQC v.0.11.9 (Andrews 2010). Based on the results, low-reliability base sites and reads were eliminated per sample using fastp v.0.22.0 (Chen et al. 2018). For instance, in *HNSc*, 13 bases from the 5′ end (due to base content mismatch between A/T or G/C), one base from the 3′ end, and poly-X tail of ≥10 bases were trimmed. Bases with <Q30 were trimmed from both the ends. A window of four bases was slid from the 5′ end, and regions with an internal mean of <Q25 and subsequent sequences were excluded. Reads with >40% bases with <Q25, containing > five N sites, or <20 bp were discarded. Base correction of overlapped paired-end reads was applied. Although the number of trimmed bases owing to base content mismatch varied among the samples, the other conditions were consistent across all samples. Following QC, data quality enhancement was confirmed through reanalysis using FastQC.

### Reference Genome Preparation

At the time of our analysis, three species were available as genome resources of the genus *Vulpes*: the red fox (GCF_003160815), Tibetan sand fox (GCA_024500485), and Arctic fox (*V. lagopus* Linnaeus, 1758: GCF_018345385). The red fox genome was sequenced from crossbreeding (*F*_1_) between ethologically aggressive and tamed lineages selectively bred on a Russian experimental farm (Kukekova et al. 2018). This red fox genomic data is appropriate as a reference, given its ∼94× sequencing depth. However, although the assemblies of Tibetan sand and Arctic foxes were at the chromosome level (Peng et al. 2021; Lyu et al. 2022), the red fox genome was divided into 82,423 scaffolds. Using a genome from another species directly as a pseudoreference may introduce significant bias in downstream analyses (Prasad et al. 2022). Therefore, additional scaffolding of the red fox draft genome was required to use as a mapping reference. Rando et al. (2018) assembled these red fox scaffolds into 40 pseudomolecules; however, because the study used outgroup genomes from the domestic dog and cat (*Felis silvestris catus* Linnaeus, 1758), distantly related to the red fox, the number of fragments still differed from that of actual red fox chromosomes.

In the mitogenome phylogeny, the red fox is more closely related to the Tibetan sand fox than the Arctic fox (Zhao et al. 2016). Additionally, the karyotype of the red fox (2*n* = 34 + B) more resembles that of the Tibetan sand fox (2*n* = 36) than the Arctic fox (2*n* = 50) (Mäkinen 1985; Jaszczak et al. 1987; Lyu et al. 2022). Thus, we defined the 18 chromosome assemblies from the Tibetan sand fox as a pre-reference genome and assigned the 82,423 scaffolds from the red fox to them using RagTag v.2.1.0 (Alonge et al. 2022). Minimap2 v.2.24 (Li, 2018) was used as a mapping aligner. The unplaced scaffolds were concatenated into a single fragment. The 18 synthesized pseudomolecules of the red fox, syntenic to chromosomes of the Tibetan sand fox, were used as a reference genome in this study. Furthermore, a mitogenome sequence included in GCF_003160815 was added to the reference dataset.

### Variant Calling

Mapping of the post-QC paired-end reads of each sample to the reference genome was executed using BWA-MEM2 v.2.2.1 (Vasimuddin et al. 2019). Subsequently, the mark up of PCR-duplicates for deduplication, data sorting, and compression from SAM to BAM were performed using the MarkDuplicatesSpark command in GATK v.4.4.0.0 (Broad Institute, Cambridge, MA, U.S.A.; Van der Auwera and O'Connor 2020).

To detect the variants, a gVCF file was generated per sample from BAM using GATK HaplotypeCaller. The gVCF files from all samples were integrated into a single database using GATK GenomicsDBImport. Joint genotyping was conducted on this database using GATK GenotypeGVCFs to generate a multi-sample VCF. After separating the SNPs and indels using GATK SelectVariants, hard-filtering based on different thresholds between them was applied using GATK VariantFiltration. For SNPs, Qual < 30.0, QualByDepth < 2.0, FisherStrand > 60.0, StrandOddsRatio > 4.0, RMSMappingQuality < 40.0, MappingQualityRankSumTest < −12.5, and ReadPosRankSumTest < −8.0 were set as filters, following the recommendation of GATK technical documentation (https://gatk.broadinstitute.org/hc/en-us/categories/360002310591 [last accessed on June 6, 2023]). For indels, the filters were set at Qual < 30.0, QualByDepth < 2.0, FisherStrand > 200.0, StrandOddsRatio > 10.0, and ReadPosRankSumTest < −20.0. Only variants that passed all filters were written into new VCF files using GATK SelectVariants.

The calibration model for base quality score recalibration (BQSR) was constructed using filtered variants as prior information with GATK BaseRecalibrator. The recalibration table was applied to the BAM of each sample using GATK ApplyBQSR, adjusting the *Q*_base_ downward. Procedures from variant calling to filtration were re-executed using the corrected BAM to generate the final VCF files.

### SNP Analysis

Variant statistics among samples were calculated using the vcf-compare command in VCFtools v.0.1.16 (Danecek et al. 2011) to draw a Venn diagram. The mapping coverage and mean depth of each sample were determined from post-BQSR BAM using the coverage command of SAMtools v.1.17 (Danecek et al. 2021), and a sex-chromosomal pseudomolecule was identified based on the depths.

Following the extraction of biallelic SNPs on 17 autosomal pseudomolecules from VCF using GATK SelectVariants, LD-pruning was conducted with a window size of 50 SNPs, step size of five SNPs, and threshold of *r^2^* > 0.5 (variance inflation factor [VIF] > 2.0) using the -indep option in PLINK v.1.90b6.21 (Purcell et al. 2007). Based on SNPs after LD-pruning, PCA and computing of the *H_O_* were performed using PLINK -pca and -het. Additionally, RoH regions on the genomic structure were detected using PLINK -homozyg. RoH are observed more frequently and longer in populations affected by bottlenecks and inbreeding. Continuous homozygous sequences with >1 Mbp, including >100 SNP loci (at least one SNP per 50 kbp), were considered RoH.

To evaluate nucleotide diversity, *θ_W_* was computed per sample based on the SFS using ANGSD v.0.940 (Korneliussen et al. 2014). Site allele frequency likelihood was calculated from post-BQSR BAM file using ANGSD doSaf 1. The reference genome was used as an ancestral character, and the folded SFS was estimated using ANGSD realSFS. Subsequently, *θ_W_* was computed from 17 autosomal pseudomolecules using ANGSD saf2theta and thetaStat.

Ancestral clustering analysis was executed from post-LD-pruning SNPs using ADMIXTURE v.1.3.0 (Alexander et al. 2009). The ancestry rate of each sample and cross-validation error were estimated based on *K* = 2 to 4.

### Assembly and Annotation

After splitting multi-sample VCF per sample, concatenating SNP and indel files, and excluding multiallelic loci, a reference-guided assembly was conducted for each sample by applying variants to the reference genome using GATK FastaAlternateReferenceMaker. Heterozygous sites were preserved as an ambiguity (mixed-base) code defined by the International Union of Pure and Applied Chemistry (IUPAC). The constructed assemblies of 18 pseudomolecules and a mitogenome were registered in the INSD (accession number: BAABOF010000001–BAABOI010000019). Assembly statistics were assessed for the pseudomolecule sequences using AssemblyStatistics v.1.1.3 (Lin and Seaman 2023).

Pairwise genomic distances among samples were determined based on 18 pseudomolecules. Hamming and MinHash distances were computed using Skmer v.3.3.0 (Sarmashghi et al. 2019) and Mash v.2.3 (Ondov et al. 2016), respectively, with a *k*-mer size of 31 and sketch size of 10 million.

Gene prediction and functional annotation were executed through the modular open-source genome annotator (MOSGA) web pipeline (https://mosga.mathematik.uni-marburg.de/index.php [last accessed on July 15, 2023]) (Martin et al. 2020) for 18 pseudomolecules of each sample. Repeats were identified and soft-masked using Red (Girgis 2015). Protein-coding sequences (CDS) after messenger RNA (mRNA) splicing were predicted ab initio, using Augustus (Stanke and Waack 2003) and a trained model constructed for the human (*Homo sapiens* Linnaeus, 1758). Sequences of rRNA and tRNA were detected using Barrnap (Seemann 2018) and tRNAscan-SE 2 (Chan et al. 2021), respectively. Annotation of functional genes was performed through a homology search against Swiss-Prot (Bairoch and Apweiler 2000) using Diamond (Buchfink et al. 2015). A generated GFF file was applied to the assembly per sample to extract DNA sequences of transcripts (including protein-coding gene, rRNA, and tRNA) into FASTA using GffRead v.0.12.7 (Pertea and Pertea, 2020) (Data TS).

### Genomic Phylogenetic Analysis

For phylogenetic estimation, orthology analysis of the transcripts on the 17 autosomal pseudomolecules was conducted to eliminate paralogs resulting from gene duplication. Orthogroup, representing gene group derived from a single ancestral gene, was identified using OrthoFinder v.2.5.5 (Emms and Kelly 2019). Orthogroups that included only single-copy transcripts per sample were considered orthologs.

Orthologous transcripts were combined and aligned to create a multiple sequence alignment (MSA) using MAFFT v.7.520 (Katoh et al. 2002). The reference genome from a continental individual on a Russian farm (Kukekova et al. 2018) was incorporated into the MSA as an outgroup because the Hondo red fox is a monophyletic group that evolved within the Japanese Archipelago, according to mtDNA phylogeny (Watanabe and Yamazaki 2024). For the nucleotide substitution model, GTR+Γ4+I was selected based on the corrected Akaike information criterion (AICc) using ModelTest-NG v.0.1.7 (Darriba et al. 2020). Subsequently, the maximum likelihood tree was estimated with a bootstrap reconstruction of 100,000 iterations using RAxML-NG v.1.2.0 (Kozlov et al. 2019).

As the transcript-based tree may be affected by adaptive mutations, we assessed an overall-SNP-based tree. Autosomal SNP loci were concatenated using vcf2phylip.py v.2.8 (Ortiz 2019). A maximum likelihood tree with ultrafast bootstrap approximation and Shimodaira–Hasegawa-like approximate likelihood-ratio test (SH-aLRT) of 10,000 times each was estimated using IQ-TREE v.2.3.5 (Minh et al. 2020), based on the MFP+ASC (automatically selected substitution model based on AICc + ascertainment bias correction) model.

Mitogenome sequences from continental individuals were collected from the INSD for phylogenetic analysis: AM181037, GQ374180, JN711443, KF387633, KJ140137, and MN122913. The corsac fox (*V. corsac* Linnaeus, 1768) was adopted as the outgroup (Zhao et al. 2016). The mitogenome was divided into 37 transcripts (13 CDS, two rRNA, and 22 tRNA), and the D-loop region was eliminated for alignment accuracy. Stop codons for *ND1* to *ND4* and *COX3*, completed by post-transcriptional modification, were adjusted to triplets. *ND6*, situated on a different strand from the other 12 CDS, was reversed-complementarily. Nucleotide substitution models were selected for each of the 63 partitions (two rRNA, 22 tRNA, and each codon position of 13 CDS) using Kakusan v.4 (Tanabe 2011). In the maximum likelihood method, the EqualRate–CodonEqualRate model was adopted based on the AICc 4. A shotgun ML search with 10,000 bootstrap resampling was run using RAxML v.8.2.10 (Stamatakis 2014). For Bayesian methods, the Proportional–CodonProportional model was selected based on the Bayesian information criterion (BIC) 4. A Markov chain Monte Carlo (MCMC) algorithm was run for 30 million generations with a sampling frequency of every 100 generations using MrBayes v.3.2.7 (Ronquist et al. 2012). After confirming the convergence of Bayesian parameters and discarding data from the first quarter as burn-in, the tree was summarized and visualized.

### Coalescent Estimation

PSMC simulation (Li and Durbin 2011) was executed based on 17 autosomal pseudomolecule assemblies to reconstruct the historical demographic dynamics of the population to which each sample belongs. The diploid consensus sequence of each individual was divided into 100-bp bins, and the presence of a heterozygous locus was scored using fq2psmcfa, implemented in the software package psmc v.0.6.5. The converted string was split for bootstrap analysis using psmc splitfa. PSMC was run for the whole genome and 100 bootstrapped split genomes. Three conditions were set for a temporal segment pattern of 64 atomic time intervals: 4+25*2+4+6 (C1: default setting used in numerous studies), 2+2*1+25*2+4+6 (C2: split the first free interval parameter to improve the resolution in the post-glacial stage), and 2*2+50*1+4+6 (C3: split middle free interval parameters to detect fine demography). The dynamics of *N_e_* was plotted using psmc_plot.pl. For time scaling, the absolute genomic mutation rate *µ* (per site/generation) was set to 4.46 × 10^−9^ based on a previous estimation of germline mutation rate using whole genomes from a parent–offspring trio of the red fox (Bergeron et al. 2023). Although the red fox usually reaches sexual maturity in one year, the generation time *g* is likely close to two years (Goddard et al. 2015; Preckler-Quisquater et al. 2024). Therefore, we adopted *g* = 2; however, results from *g* = 1 were also presented as supplements (the plot just shifts toward the more recent times). Since PSMC is sensitive to mapping depth owing to its heterozygosity-based method, FNR correction was applied to the plot from a sample with a mean depth of <30×.

MSMC simulation (Schiffels and Durbin 2014), a model that extends PSMC to multiple individuals, was performed to estimate the dynamics of ancestral population size and demographic differentiation. In this study, the Hondo red fox group per island was considered a single population. However, since the genomes were not phased, the analysis was conducted on a per-sample basis (*HNSc* and *HNSw* were not integrated). With one diploid genome per population, phasing is likely less problematic, as there are only two haplotypes (a specific pair). Mapping reads on 17 autosomal pseudomolecules of each sample were converted from post-BQSR BAM to a pileup format using the mpileup command of BCFtools v.1.9 (Danecek et al. 2021). Subsequently, SNPs and mask data were called using the BCFtools call and bamCaller.py from the msmc-tools v.2.1.4. Input files for MSMC were generated per pseudomolecule using msmc generate_multihetsep.py. Bootstrapping was performed by synthesizing 5-Mbp duplicate-allowed random fragments to generate 17 virtual pseudomolecules of 100 Mbp each using msmc multihetsep_bootstrap.py; this was repeated 20 times. The chronological coalescence rates in intra-populational and inter-populational genomes were computed using msmc2_Linux. The time-segment pattern was set to 10*1+22*2+4+6 based on a previous optimization by Quinn et al. (2024) for improving the resolution during the post-glacial stage by breaking the first time segment. The *µ* and *g* were set to the same conditions as those of PSMC. The relative cross-coalescence rate was calculated by dividing twice the inter-populational coalescence by the sum of each intra-populational coalescence (2Λ_01_/[Λ_00_ + Λ_11_]) using msmc combineCrossCoal.py. This ratio approaches zero when a coalescence of inter-population ceases, reflecting a demographic divergence. The results of the demographic simulation and population separation history estimation were plotted using R v.4.3.2 (R Core Team, Vienna, Austria).

Since PSMC-based models are weak at detecting demography within ∼10 ka, we executed the SMC++ (Terhorst et al. 2017) algorithm based on SFS and LD information. The input files were generated from VCF per autosomal pseudomolecule using vcf2smc in SMC++ v.1.15.4. The composite likelihood approach was applied to the Honshu population, including the two individuals (SMC++ is not affected by unphased genomes), by varying distinguished lineages. Bootstrap permutations of 20 times were performed using the SMC++ chunk command. Fitting population size history to data and automatic optimization of model parameters based on cross-validation were performed using the SMC++ cv command. The *µ* and *g* were set to the same values as those of PSMC. Additionally, joint frequency spectrum between populations was generated using the vcf2smc, and the joint demography was estimated using the SMC++ split command. The split time was calculated by 2*N_0_* × coalescent split value × *g*. The distribution of split times from 20 bootstrap dataset was represented as a violin plot using R.

To estimate historical fluctuations in the inter-populational migration rate, MiSTI (Shchur et al. 2022) simulation was executed. As this new program has few examples of use, we followed the methodology of Sarabia et al. (2021). The multi-sample joint SFS was generated using ANGSD doSaf and realSFS and converted using ANGSDSFS.py in MiSTI v.0.2.3. For scaling, time discretization points of PSMC C1 results were merged between two individuals using calc_time.py. The inference was repeated 50 times by varying the split time index from 1 to 50, and the log-likelihood of each split scenario was computed using MiSTI.py. We set 24 migration bands: time interval indexes 0–2, 3–5, 6–8, 9–11, 12–14, 15–17, 18–20, 21–25, 26–30, 31–35, 36–40, and 41–50 (until ∼150 ka) and these bidirectional migration sources. The migration rate was automatically optimized. For the split scenario with the highest log-likelihood, the optimized migration rate of each band was plotted using R.

## Supplementary Material

evaf152_Supplementary_Data

## Data Availability

Raw sequencing reads and genome assemblies (including 18 pseudomolecules and a mitogenome) of four Hondo red foxes are available in the INSD (BioProject: PRJDB17515, BioSample: SAMD00738003–SAMD00738006, raw read FASTQ: DRR530053–DRR530056, and assembly FASTA: BAABOF010000001–BAABOI010000019 [*HNSc*: BAABOI, *HNSw*: BAABOH, *SKK*: BAABOF, and *KS*: BAABOG]). The predicted transcript sequences for each genome are available online as supplementary data for this paper.
