# Supplementary material for: Inter-Island Whole-Genome Comparison Reveals Micro-Evolutionary Dynamics of the Red Fox, Stimulated Through Post-Glacial Sea-Level Alterations
Source: Genome Biol Evol. 2025 Aug 21;17(8):evaf152. doi: 10.1093/gbe/evaf152 (PMC12368961; doi:10.1093/gbe/evaf152)

Supplementary Figure S1

Base quality score distribution of sequencing data from four Hondo red fox samples. Horizontal and vertical axes are the mean base quality score Q_{base} of a read and the number of reads with that mean Q_{base}, respectively. Samples are identified by color. The upper and lower rows are reads 1 (A and C) and 2 (B and D) of paired-ends, and the left and right columns are raw (A and B) and post-QC (C and D) data, respectively.

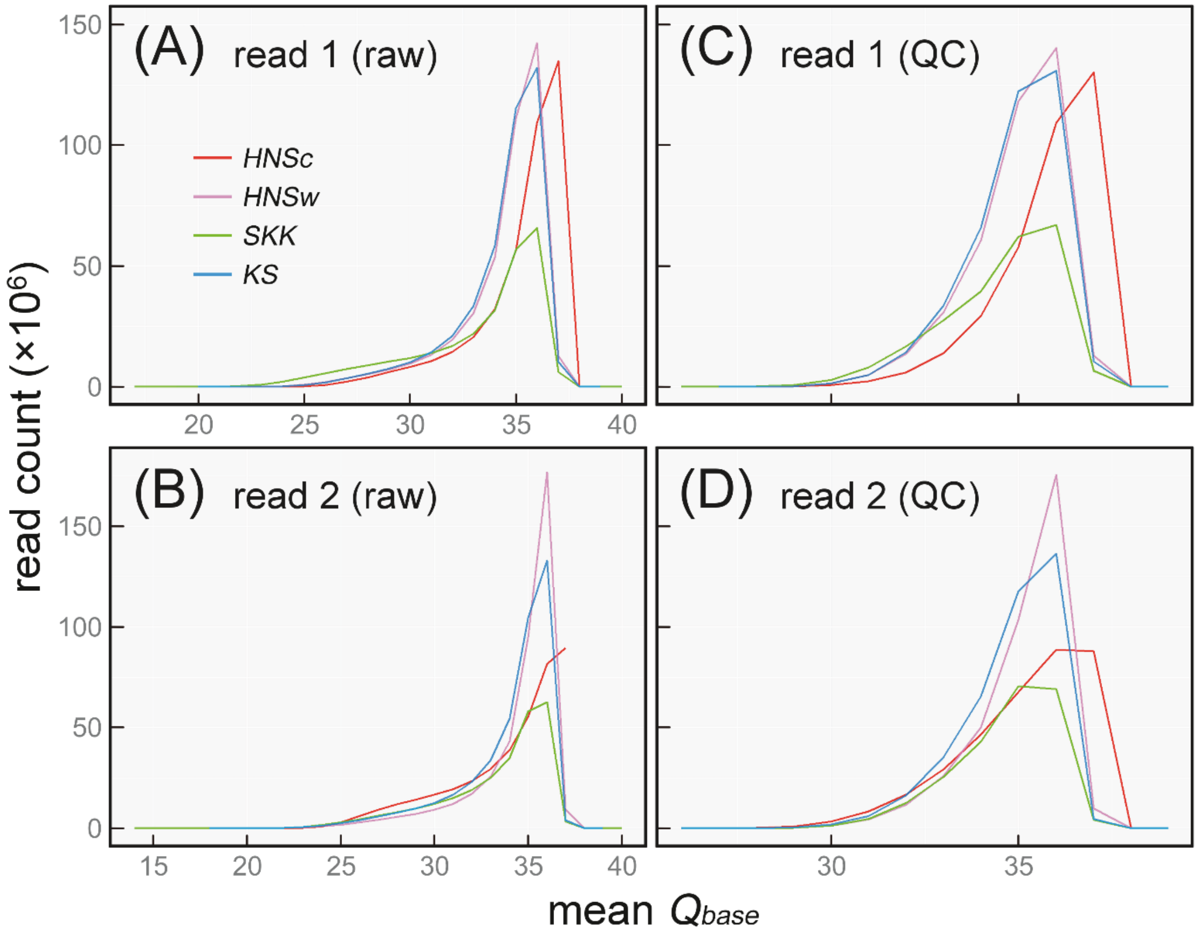

Supplementary Figure S2

Venn diagram of variant statistics among the four Hondo red fox samples. The denominator and numerator are the number of sharing variants and SNPs, respectively.

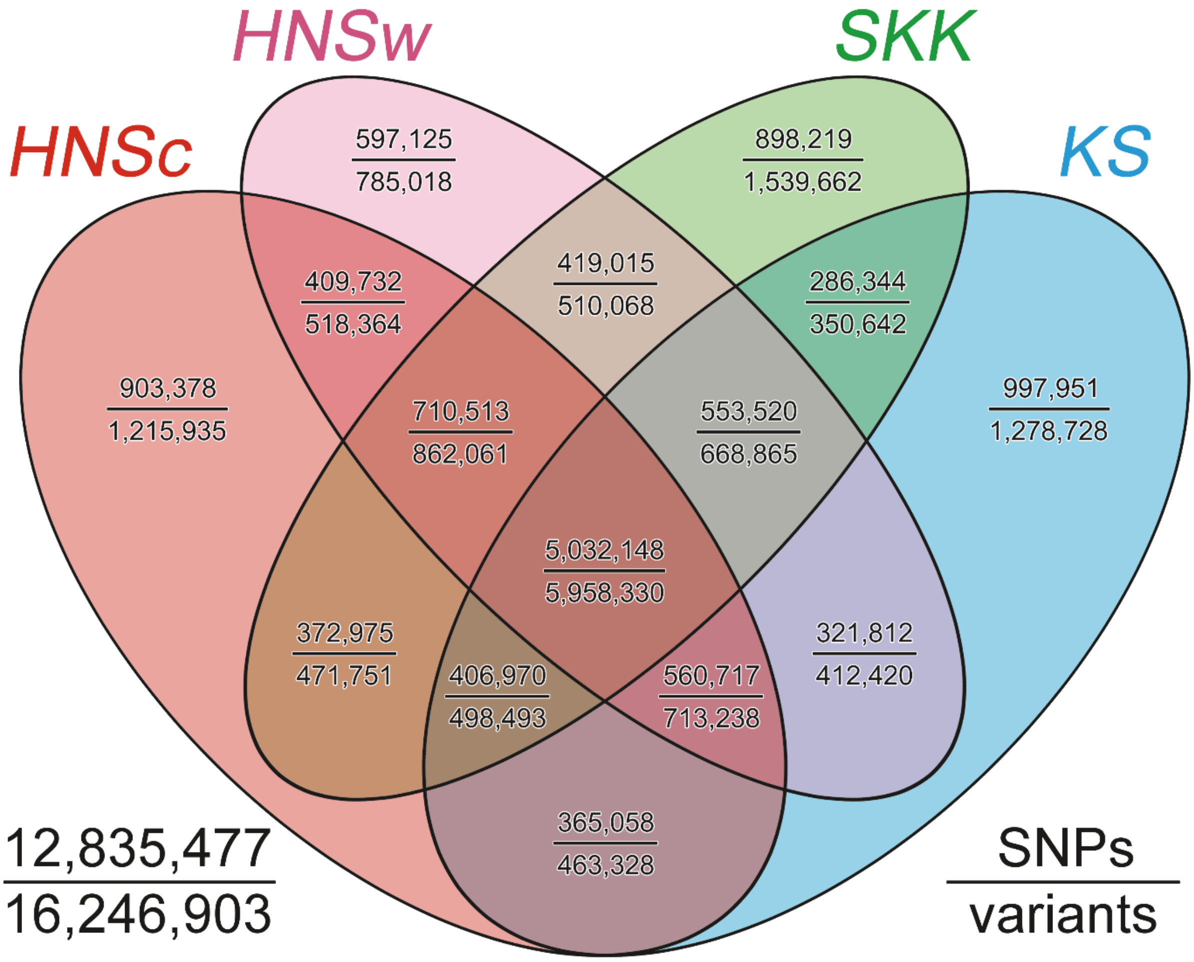

Supplementary Figure S3

Phylogenetic tree of four Hondo red fox samples based on a concatenated sequence of 7,228,522 SNP sites on 17 autosomal pseudomolecules. It was estimated by the maximum likelihood method using IQ-TREE v.2.3.5. However, because significant differences were detected in base composition among sequences by χ^2 test (P < 0.05), this may not be a reliable analysis. Support rate (ultrafast bootstrap approximation / SH-like approximate likelihood-ratio test) is denoted above the branch. The scale bar represents the measure of genetic distance.

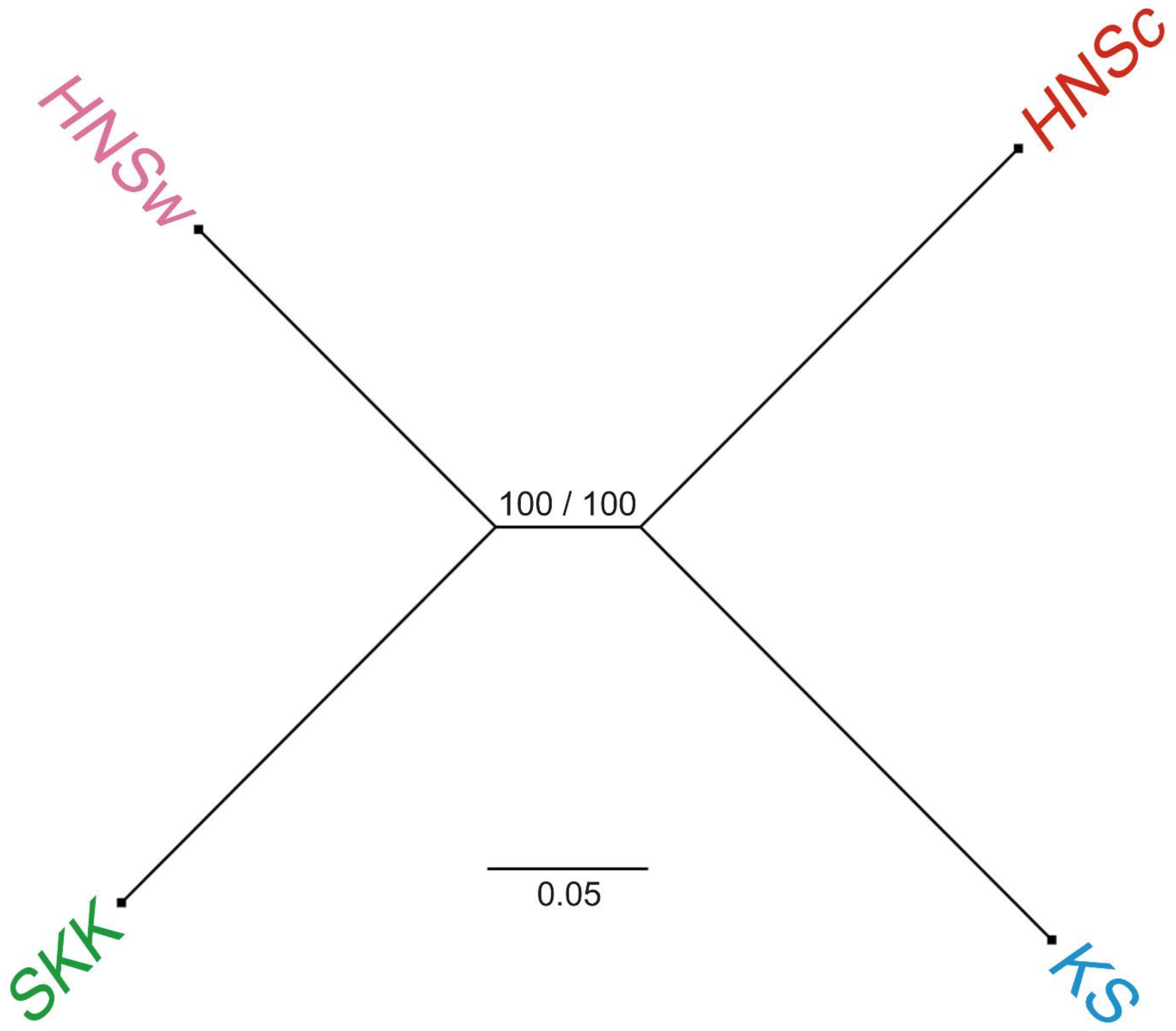

Supplementary Figure S4

PCA plot of four Hondo red fox samples based on biallelic SNPs on 17 autosomal pseudomolecules. The upper (A–C) and lower (D–F) rows were calculated from overall (12,248,003 loci) and post-LD-pruning (489,033 loci) SNPs, respectively. Samples are identified by color. The contribution rate of each PC axis is presented in parentheses.

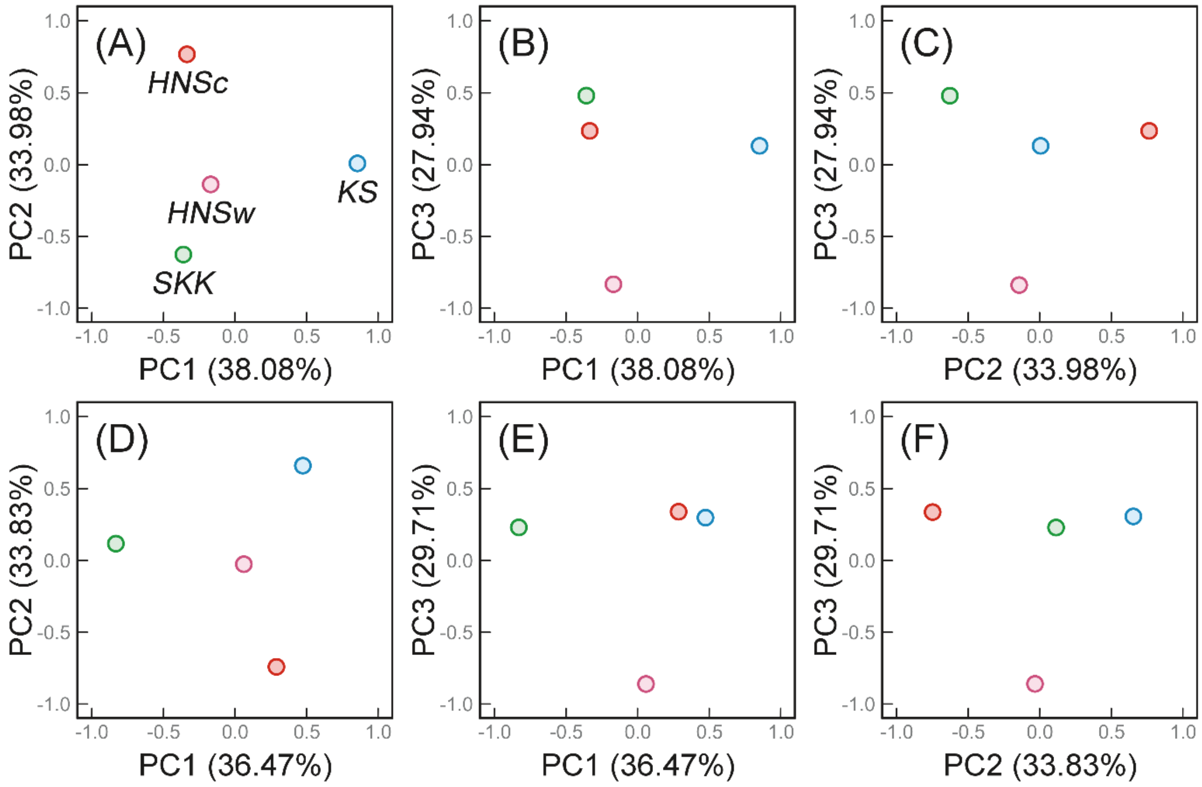

Supplementary Figure S5

Ancestral clustering analysis of four Hondo red fox samples based on 489,033 post-LD-pruning SNPs on 17 autosomal pseudomolecules. (A) Cross-validation test to determine the number of ancestral clusters *K*. (B) Ancestry of each sample based on *K* = 2–4. Assessment of admixture was not possible due to the small sample size.

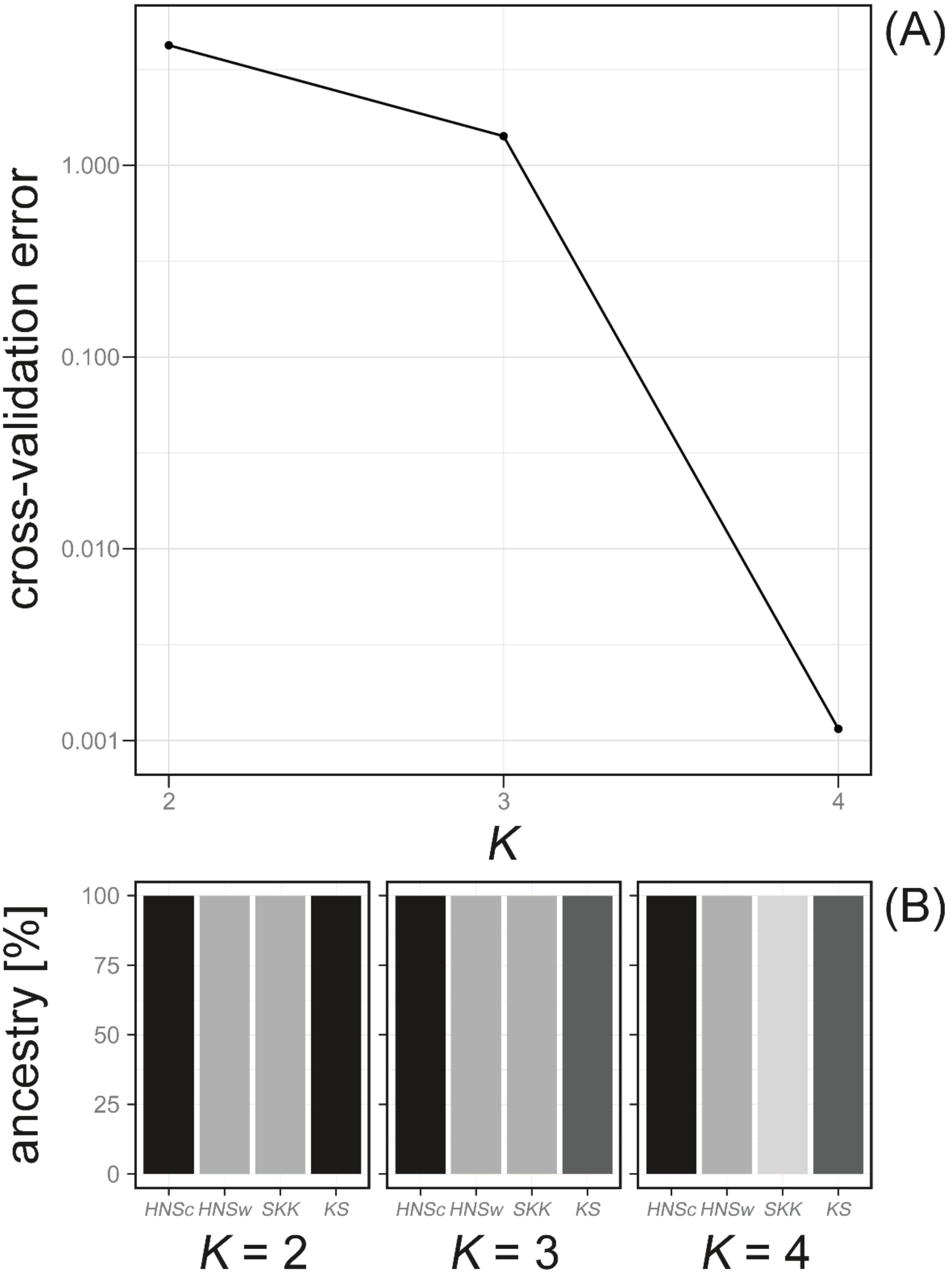

Supplementary Figure S6

(A–D) Bootstrap iteration of PSMC under time segment condition C1. Horizontal and vertical axes are a logarithmic timeline and effective population size N_e, respectively. Four Hondo red fox samples are coded by line color. The dark line is based on the whole-genome, whereas the 100 light lines are based on bootstrap permutations. (E) PSMC C1 plots for generation time $g = 1$.

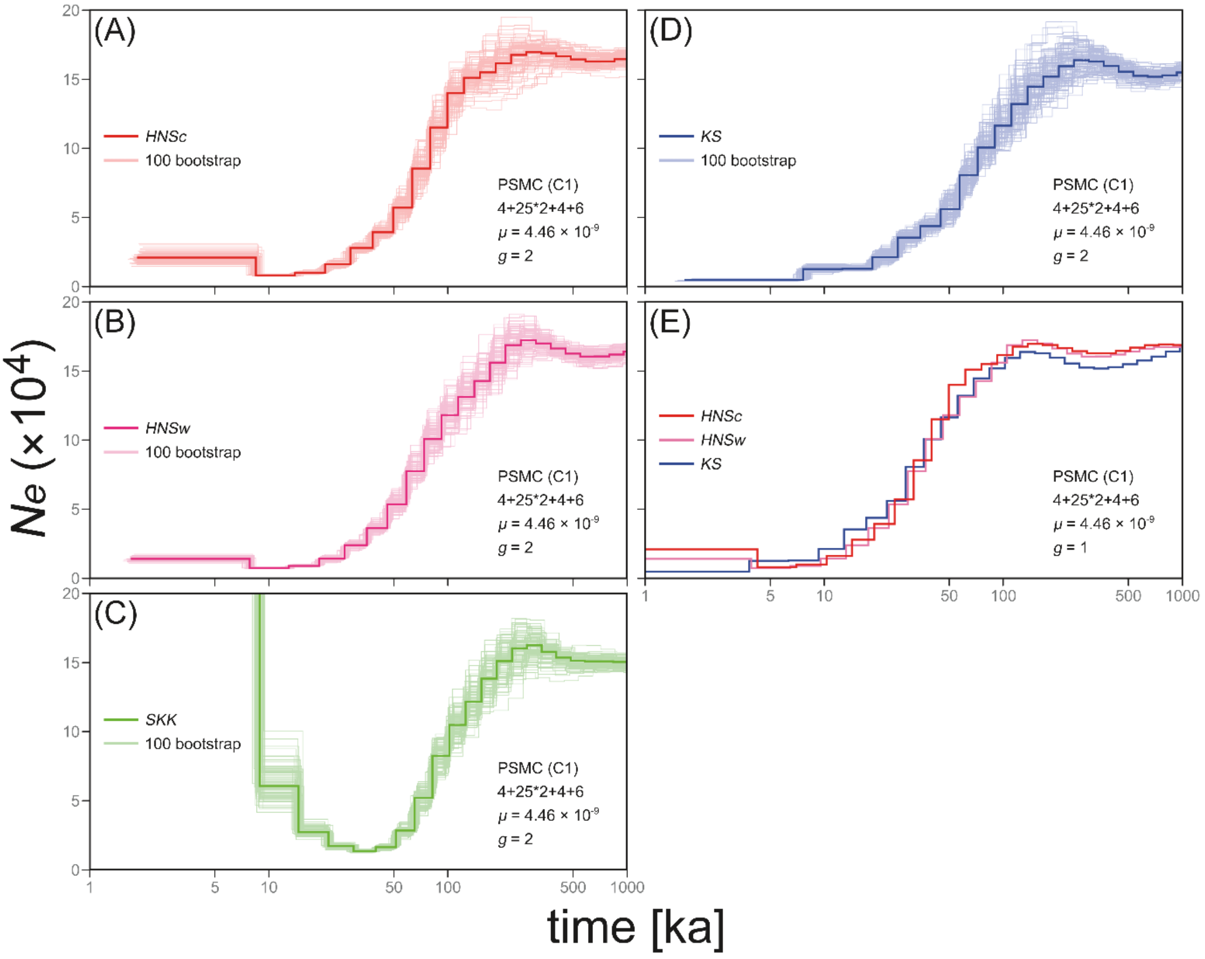

Supplementary Figure S7

(A–C) Bootstrap iteration of PSMC under time segment condition C2. Horizontal and vertical axes are a logarithmic timeline and effective population size N_e, respectively. Three Hondo red fox samples are coded by line color. The dark line is based on the whole-genome, whereas the 100 light lines are based on bootstrap permutations. (D) PSMC C2 plots for generation time g = 1.

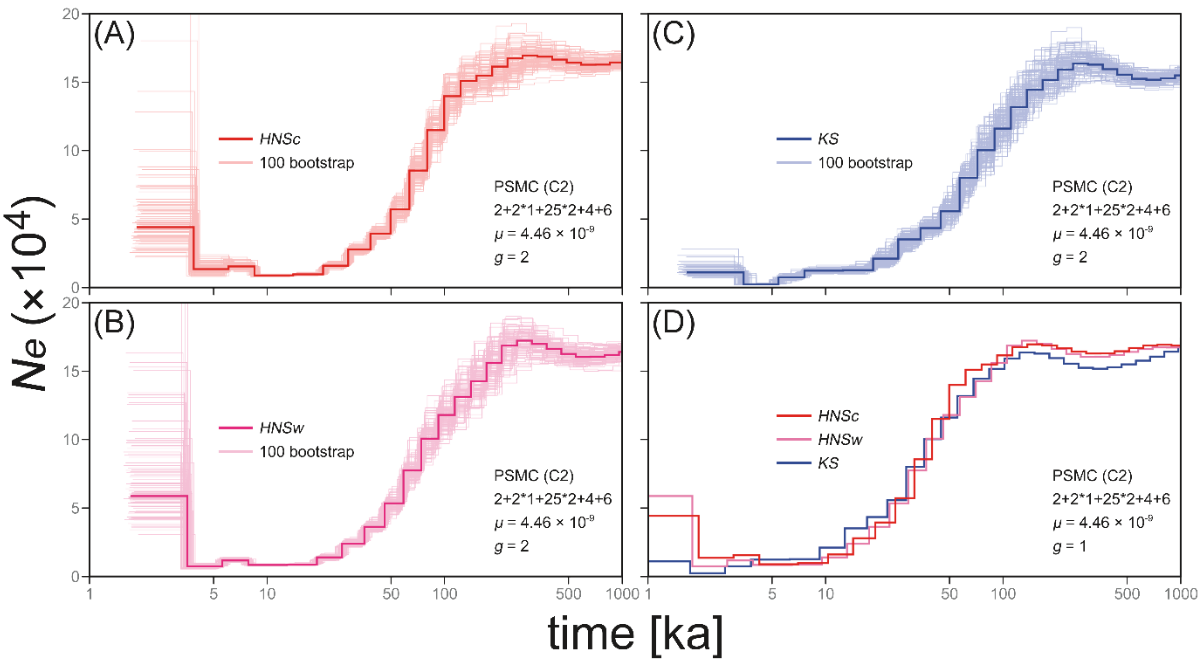

Supplementary Figure S8

(A–C) Bootstrap iteration of PSMC under time segment condition C3. Horizontal and vertical axes are a logarithmic timeline and effective population size N_e, respectively. Three Hondo red fox samples are coded by line color. The dark line is based on the whole-genome, whereas the 100 light lines are based on bootstrap permutations. (D) PSMC C3 plots for generation time g = 1.

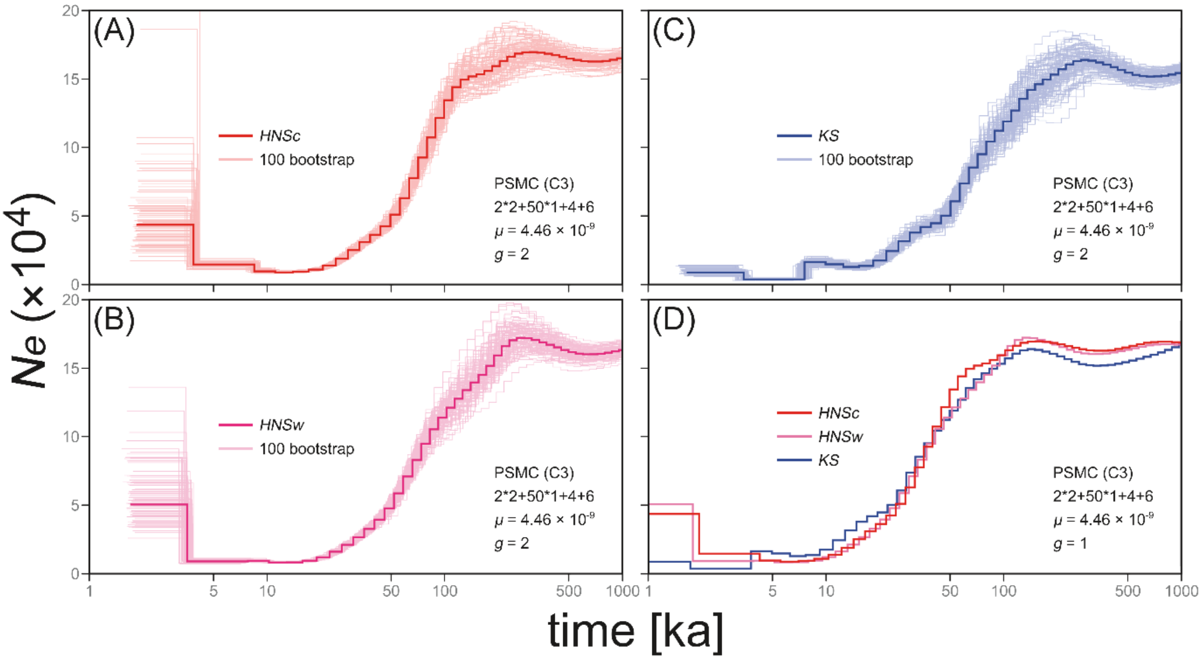

Supplementary Figure S9

Bootstrap iteration of (A–C) MSMC and (D and E) SMC++. Horizontal and vertical axes are a logarithmic timeline and effective population size N_e, respectively. Three Hondo red fox samples or two populations are coded by line color. The dark line is based on the whole-genome, whereas the 20 light lines are based on bootstrap permutations.

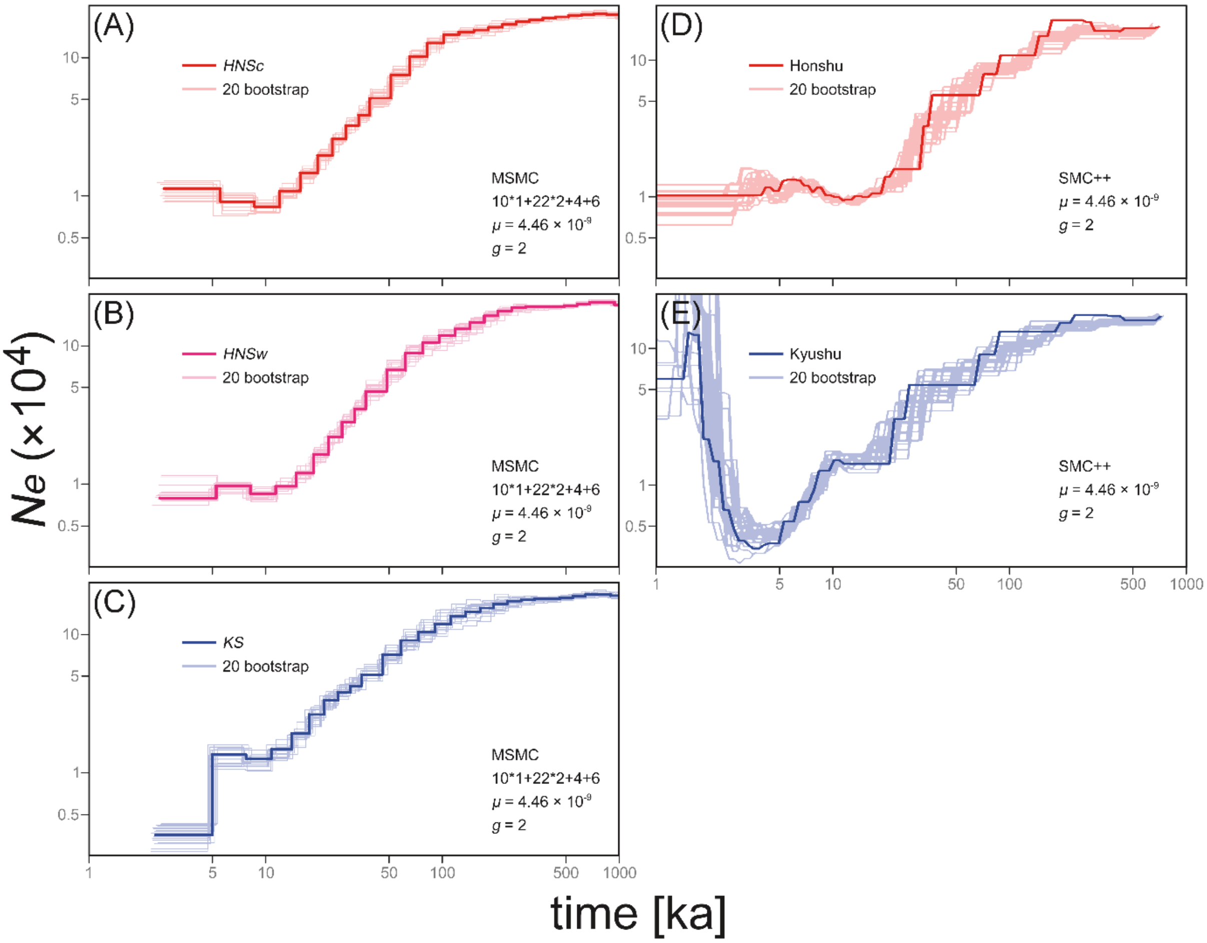

Supplement: evaf152_Supplementary_Data [file evaf152_supplementary_data.zip › File S2.pdf]
